# Melatonergic signalling instructs transcriptional inhibition of IFNGR2 to lessen interleukin‐1β‐dependent inflammation

**DOI:** 10.1002/ctm2.716

**Published:** 2022-02-20

**Authors:** Yaoyao Xia, Qingzhuo Zhang, Yuyi Ye, Xiaoyan Wu, Fang He, Yuanyi Peng, Yulong Yin, Wenkai Ren

**Affiliations:** ^1^ State Key Laboratory for Conservation and Utilization of Subtropical Agro‐Bioresources Guangdong Laboratory of Lingnan Modern Agriculture Guangdong Provincial Key Laboratory of Animal Nutrition Control National Engineering Research Center for Breeding Swine Industry College of Animal Science South China Agricultural University Guangzhou China; ^2^ College of Veterinary Medicine Southwest University Chongqing China; ^3^ Institute of Subtropical Agriculture Chinese Academy of Sciences Changsha China

**Keywords:** HSF1, IFNGR2, IL‐1β, macrophage, melatonin

## Abstract

**Background:**

Immunotransmitters (e.g., neurotransmitters and neuromodulators) could orchestrate diverse immune responses; however, the elaborated mechanism by which melatonergic activation governs inflammation remains less defined.

**Methods:**

Primary macrophages, various cell lines, and *Pasteurella multocida* (PmCQ2)‐infected mice were respectively used to illustrate the influence of melatonergic signalling on inflammation in vitro and in vivo. A series of methods (e.g., RNA‐seq, metabolomics, and genetic manipulation) were conducted to reveal the mechanism whereby melatonergic signalling reduces macrophage inflammation.

**Results:**

Here, we demonstrate that melatonergic activation substantially lessens interleukin (IL)‐1β‐dependent inflammation. Treatment of macrophages with melatonin rewires metabolic program, as well as remodels signalling pathways which depends on interferon regulatory factor (IRF) 7. Mechanistically, melatonin acts via membrane receptor (MT) 1 to increase heat shock factor (*Hsf) 1* expression through lowering the inactive glycogen synthase kinase (GSK3) β, thereby transcriptionally inhibiting interferon (IFN)‐γ receptor (IFNGR) 2 and ultimately causing defective canonical signalling events [Janus kinase (JAK) 1/2‐signal transducer and activator of transcription (STAT) 1‐IRF7] and lower IL‐1β production in macrophages. Moreover, we find that melatonin amplifies host protective responses to PmCQ2 infection‐induced pneumonia.

**Conclusions:**

Our conceptual framework provides potential therapeutic targets to prevent and/or treat inflammatory diseases associating with excessive IL‐1β production.

## Graphical abstract

Melatonergic signalling activated by melatonin causes a transcriptional downregulation of IFNGR2 and impairs canonical signalling events (JAK1/2‐STAT1‐IRF7) in LPS/IFN‐γ‐stimulated macrophages through MT1‐GSK3β (inactive)‐Hsf1 axis, thereby reducing IL‐1β production.

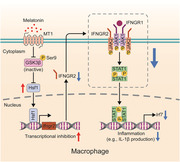

## 1 INTRODUCTION

Extracellular functions of multitudinous substances and their derivatives are clear as transmitters between nerve cells and/or immune cells, functioning as immunotransmitters (e.g., neurotransmitters and neuromodulators).^1^ Notably, the nervous system physiologically contributes to direct immune homeostasis and inflammatory diseases through various immunotransmitters. The examples include dopamine in the functions of myeloid and lymphoid immune cells;^2^, ^3^ acetylcholine in host defense against intestinal infections;^4^, ^5^ serotonin in platelet activation,^6^ macrophage polarization,^7^, ^8^ and T cell activation;^9^ as well as γ‐aminobutyric acid (GABA) in the macrophage function.^10^ Nevertheless, the roles and delicate mechanisms for these mediators in orchestrating immune responses and/or inflammatory diseases are still not fully understood.

The interleukin (IL)‐1 family cytokines (mainly IL‐1β) are highly responsible for host defense against pathogens.^11^, ^12^ Indeed, many inflammatory cells, including the macrophages, could secrete large amount of mature IL‐1β.^13^, ^14^ Noteworthy, the prolonged production of IL‐1β triggers imbalance of the immune system, leading to a spectrum of inflammatory diseases; thus, it is of great importance to precisely fine‐tune IL‐1β production. Generally, the production of mature IL‐1β (17 kD) depends on the processing of immature pro‐IL‐1β (31 kD) followed by proteolytic cleavage involving caspase‐1.^15^ Many canonical/non‐canonical inflammasome‐mediated regulators (e.g., NOD‐like receptor NLRP3, caspase‐8, and caspase‐4/5/11)^16^, ^17^, ^18^ and/or inflammasome‐independent modulators (e.g., cathepsin C/G)^19^, ^20^ are involved in the processing of IL‐1β. Whereas, whether other contributors (e.g., signal transducers and/or transcriptional regulators) could determine IL‐1β production remain worth exploring.

Melatonin (a ubiquitous neuromodulators found in various organisms) is synthesized from serotonin by serotonin *N*‐acetyltransferase and hydroxyindole *O*‐methyl transferase.^21^ Indeed, melatonin is highly pleiotropic and plays numerous physiological functions, like the regulation of circadian rhythms,^22^ inhibiting pathogens,^23^ and scavenging free radicals.^24^ Since melatonin is reported to be produced by immune cells, and a growing body of evidence has shown that peripheral immune cells express functional melatonergic components, including membrane receptors (MT1/MT2) and enzymes involved in melatonin synthesis,^25^ the melatonin's actions in immunomodulation deserve particular attention.^26^ For example, melatonin blocks retinoid‐related orphan nuclear receptor (ROR) γt expression and T helper (Th) 17 cell differentiation^27^, ^28^ and orchestrates the phenotype polarization of macrophages.^21^ Moreover, melatonin has been demonstrated to attenuate organ failures and macrophage‐mediated inflammatory responses in vivo through inhibiting inflammasome activation (including NLRP3).^29^, ^30^ However, the mechanism whereby melatonergic signalling activated by melatonin influences inflammation (especially for IL‐1β production) is still needed to be comprehensively revealed.

Given that pro‐inflammatory macrophages produce large amounts of IL‐1β, herein, we use murine macrophage as model to investigate the intrinsic mechanism underlying melatonergic activation in governing IL‐1β production. We demonstrate that melatonin highly affects transcriptional and metabolic events in lipopolysaccharide (LPS) plus interferon (IFN)‐γ‐activated macrophages, especially reduces IL‐1β production. Moreover, we identify MT1–glycogen synthase kinase (GSK) 3β–heat shock factor (Hsf) 1 as a vital pathway that accounts for the transcriptional inhibition of interferon (IFN)‐γ receptor (IFNGR) 2 and the suppression of subsequent pro‐inflammatory signalling cascades mediated by melatonin. Our findings provide evidence that modulation of the melatonergic system might have implications for the prevention of inflammatory diseases associating with excessive IL‐1β production.

## 2 METHODS

### 2.1 Bacterial strains and culture

*P. multocida* CQ2 (PmCQ2) was kindly provided by Prof. Y. Y. Peng (Southwest University, Chongqing, China) and was cultured in Martin's broth agar supplemented with 5% horse serum at 37°C.

### 2.2 Cell lines

ANA‐1 cells were kindly provided by Dr. Y. X. Liao (Yangzhou University, Yangzhou, Jiangsu, China) and were cultured in complete DMEM [10% fetal bovine serum (FBS)+1% penicillin/streptomycin]. Cells were polarized into pro‐inflammatory macrophages by LPS (1 μg/ml) plus rmIFN‐γ (20 ng/ml) combined with or without melatonin treatment (1 mM) for 12 h or left unstimulated.

THP‐1 cells were kindly provided by Dr. J. L. Duan (Tongji University, Shanghai, China) and were cultured in the complete RPMI 1640 medium containing 10 mM HEPES, 2 mM glutamine, and 50 μM β‐mercaptoethanol. Cells were primed with 200 ng/ml PMA for 6 h and were polarized into pro‐inflammatory macrophages by LPS (100 ng/ml) plus rhIFN‐γ (20 ng/ml) combined with or without melatonin treatment (1 mM) for 24 h or left unstimulated.

### 2.3 Mice

Six‐ to eight‐week‐old and age‐ and gender‐matched Institute of Cancer Research (ICR) mice (obtained from SLAC Laboratory Animal Center, Changsha, China) were used for all experiments.

### 2.4 Murine primary peritoneal exudate macrophages and bone marrow‐derived macrophages

Peritoneal exudate macrophages (PEMs) and bone marrow‐derived macrophages (BMDMs) were obtained according to our previous study.^31^ Briefly, for PEMs, after the injection of 4% thioglycolate (2– 4 days later), PEMs were flushed from murine peritoneal cavity with phosphate buffer saline (PBS) and cultured in complete DMEM for adherent purification. Subsequently, PEMs were polarized into pro‐inflammatory macrophages with LPS (1 μg/ml) plus rmIFN‐γ (20 ng/ml) combined with or without melatonin treatment (1 mM) for 12 h or into anti‐inflammatory macrophages with rmIL‐4 (20 ng/ml) combined with or without melatonin treatment (1 mM) for 12 h or left unstimulated.

For BMDMs, murine bone marrow cells were first flushed from tibias and femurs with ice‐cold RPMI 1640 medium followed by culturing in complete RPMI 1640 medium containing macrophage colony‐stimulating factor (M‐CSF) (50 ng/ml) for 7 days to generate BMDMs. Subsequently, BMDMs were polarized into pro‐inflammatory macrophages with LPS (100 ng/ml) plus rmIFN‐γ (20 ng/ml) combined with or without melatonin treatment (1 mM) for 24 h or left unstimulated.

### 2.5 PmCQ2 infection

Mice were pretreated with melatonin (30, 60, and/or 120 mg/kg BW) (i.p.) or 10% (v:v) dimethyl sulfoxide (DMSO) (i.p.) for consecutive 7 days. Subsequently, mice were infected with PmCQ2 (i.p.) at a dose of 2.2 × 10^6^ CFU/ml in accordance with our previous study.^31^ Mice were killed at 12 h, 16 h, 24 h, and/or 32 h post infection to collect serum and lung for further analysis. For validation of the functions of HSF1 and STAT1 in vivo, mice were pretreated with melatonin (60 mg/kg BW) (i.p.) or 10% (v:v) DMSO (i.p.) for consecutive 7 days. Then, mice were anesthetized and intranasally administered (i.n.) HSF1 siRNA (2 mg/kg BW) or an equivalent dose of negative control (NC) siRNA at 24 h prior to the next processing. Subsequently, melatonin‐treated mice were treated with 800 mg/kg BW Fludarabine or 10% (v:v) DMSO (i.p.). After 4 h later, all mice were infected with PmCQ2 (i.p.) at a dose of 2.2 × 10^6^ CFU/ml and were killed at 12 h post infection to collect serum and lung which were then stored at −80°C until further analysis.

### 2.6 Cell proliferation analysis

Cell proliferation was detected through the CCK‐8 method as per the manufacturer's recommendation (Dojindo).

### 2.7 Cell apoptosis analysis

FITC Annexin V/PI apoptosis detection kit was used to assay cell apoptosis as per the recommended protocols (BD Biosciences, San Diego, CA, USA) followed by the flow cytometry analysis.

### 2.8 Enzyme‐linked immunosorbent assay (ELISA)

The culture supernatants of cells and serum and lung lysates of mice were collected to detect the levels of pro‐inflammatory and/or anti‐inflammatory cytokines (e.g., IL‐1β, TNF‐α, and IL‐10) using ELISA kits as recommended by the manufacturers.

### 2.9 Quantitative Reverse Transcription‐PCR (RT‐qPCR) analysis

Total RNA from homogenized lung or cell was extracted in accordance with the protocol of TRIzol reagent (Invitrogen, USA) and then quantified with Nanodrop 2000. RNA was reverse transcribed to cDNA using Primer Script™ RT reagent Kit based on the protocols (Takara, Qingdao, China). RT‐qPCR was performed via SYBR Green on the Quant Studio 6 Real‐Time PCR System (Thermo Fisher Scientific, America). Fold change was assessed through the 2^−ΔΔ^ *^Ct^* method using β‐actin or GAPDH for normalization. The primers were listed in Table S1.

### 2.10 Immunoblot analysis

Cells were lysed in radio immunoprecipitation assay (RIPA) buffer supplemented with protease and phosphatase inhibitors. The protein level was detected by bicinchoninic acid (BCA) protein assay kits. To investigate Caspase‐1 p10 and IL‐1β p17 release, cell culture supernatants were precipitated as per the previous studies’ recommendations.^32^, ^33^ After separating by 10%–12% sodium dodecyl sulfate (SDS) ‐polyacrylamide gel electrophoresis (PAGE) electrophoresis, proteins were transferred onto a polyvinylidene fluoride (PVDF) membrane followed by blocking with 5% bull serum albumin (BSA) tris‐buffered saline with tween‐20 (TBST) for 1 h. Then, the bands were incubated with primary and secondary antibodies, and visualized via electrochemiluminescence (ECL) reagent. The signal density of the film was viewed and analysed through using AlphaImager 2200 software.

### 2.11 Immunofluorescence staining

For immunofluorescence (IF) staining, cells were fixed with 4% paraformaldehyde (PFA) and blocked with PBS containing 5% BSA, and were incubated with primary and secondary antibodies. The nucleus was stained with 2‐(4‐amidinophenyl)‐6‐indolecarbamidine dihydrochloride (DAPI). Stained cells were observed using a confocal fluorescence microscope (Zeiss) and data were analysed using ZEN microscope imaging software.

### 2.12 Mitochondrial biomass

After the appropriate treatment, cells were incubated with a pre‐warmed (37°C) staining solution containing 100 nM MitoTracker Red CMXRos for 30 min. Fluorescence intensity was obtained by a fluorescence microplate reader with excitation/emission at 579/599 nm.

### 2.13 Mitochondrial membrane potential

Mitochondrial membrane potential (MMP) was detected using JC‐1 probe (Beyotime, China). Macrophages were seeded in a 96‐well half‐area microplate (Greiner) (4.5 × 10^4^ cells/well) and treated as described above. After washed with PBS, cells were incubated with JC‐1 staining solution for 20 min followed by washing with JC‐1 staining buffer for two times. Fluorescence intensity was obtained by a Fluorescence microplate reader with excitation/emission at 490/530 nm (monomers) or at 525/590 nm (aggregates).

### 2.14 Intracellular adenosine triphosphate (ATP) measurement

Cells were lysed in the ATP assay buffer and were centrifuged for 5 min (4°C, 12000 *g*). Then, the supernatants were collected to test ATP levels according to manufacturer's protocol (Beyotime, China).

### 2.15 Mitochondrial reactive oxygen species (ROS)

Macrophages were seeded in a 96‐well half area microplate (Greiner) (4.5 × 10^4^ cells/well) and treated as described above. Cells were washed with warm DPBS (phenol red‐free, with Ca^2+^ and Mg^2+^), then cells were incubated with 5 μM of MitoSox (Invitrogen) at 37°C for 30 min. Fluorescence intensity was obtained by a Fluorescence microplate reader with excitation/emission at 510/580 nm.

### 2.16 Cytosolic Ca^2+^ measurement

Cytosolic Ca^2+^ was assessed using Fluo‐4 AM probe (Beyotime, China). Cells were incubated with 5 μM Fluo‐4 AM in the extracellular solution at 37°C in a 5% CO_2_ incubator for 30 min, washed thrice, and incubated in the extracellular solution for an additional 20 min to allow de‐esterification. Fluorescence intensity was obtained by a Fluorescence microplate reader with excitation/emission at 488/520 nm.

### 2.17 Mitochondrial complex activity measurement

Mitochondrial complex activity (I, II, III, IV, and V) was measured using assay kit as per manufacturer's instructions (Nanjing Jiancheng Bioengineering Institute).

### 2.18 Transmission electron microscopy (TEM)

After the indicated treatments, macrophages were washed in ice‐cold PBS and fixed in 2.5% glutaraldehyde. Then, the cells were softly scraped and put into a 1.5 ml EP tube. The cells were fixed in 1% osmium tetroxide and dehydrated in a gradient ethanol series (50%, 75%, 85%, 95%, and 100%), each for 15 min. Dehydrated samples were then embedded in epoxy resin, and were sliced using an ultramicrotome (60 nm) and stained with uranyl acetate and lead citrate. All the fragments were observed under a HT‐7700 transmission electron microscope (Hitachi, Tokyo, Japan). The length of mitochondria was analysed by Image J software.

### 2.19 Gene silencing or overexpression

Genetic manipulation in macrophages was accomplished by using the Lipofectamine 2000 or 3000 transfection reagent following the protocols.

### 2.20 Seahorse assay

Real‐time measurements of oxygen consumption rate (OCR) and extracellular acidification rate (ECAR) were performed using an XFe24 Extracellular Flux Analyzer (Seahorse Agilent). The following parameters were used in the assays: seed cells 2 × 10^5^ per well, for OCR, Oligomycin 100 μM, FCCP 100 μM, and Rotenone/antimycin A 50 μM; for ECAR, glucose 1 mM, Oligomycin 1 mM, and 2‐DG 1 mM as indicated.

### 2.21 Chromatin immunoprecipitation

Chromatin immunoprecipitation (CHIP) assay was performed as the CHIP kit protocol (Abcam). Briefly, after indicated treatments, macrophages were crosslinked with 1% formaldehyde for 10 min and quenched with 125 mM glycine. Then, the samples were sonicated to generate 200– 500 bp fragments. Immunoprecipitation was performed using antibody against HSF1 (abcam). Purified DNA was used to conduct qPCR analysis by specific primer of *Ifngr2* (Table S1). Data were normalized to the corresponding DNA input control.

### 2.22 RNA‐seq analysis

After the appropriate treatment, the total RNA of PEMs was extracted as described above. Quality verification, library preparation, as well as sequencing were performed by NovoGene (Beijing). In brief, clean data were obtained by removing low‐quality reads from raw data and mapped to the reference genome. Differential expression analysis was performed in DESeq2.GO, and *P* < 0.05 was considered as the threshold for significantly differential expression. Gene ontology (GO) and Kyoto Encyclopedia of Genes and Genomes (KEGG) pathway enrichment analysis of the differently expressed genes (DEGs; fold change > 2; *P* < 0.05) were performed.

### 2.23 Metabonomics analysis

Metabolomics assays were performed at Shanghai Biotree Biotech Co. Ltd. Briefly, for 200MRM assay, 1000 μl extract solution (acetonitrile:methanol:water = 2:2:1) was added into each sample. The cell samples were homogenized at 35 Hz for 4 min and sonicated for 5 min in the ice‐water bath. The homogenization and sonication cycle was repeated for three times. Then, the samples were incubated at ‐40°C for 1 h and centrifuged at 12000 rpm for 15 min at 4°C. 800 microliter of supernatant was transferred to a fresh tube and dried in a vacuum concentrator at 37°C. Then, the dried samples were reconstituted in 100 μl of 50% acetonitrile by sonication on ice for 10 min. The constitution was then centrifuged at 12000 rpm for 15 min at 4°C, and 75 μl of supernatant was transferred to a fresh glass vial for liquid chromatography‐mass spectrometry (LC/MS) analysis.

For GC‐TOF‐MS assay, the samples were transferred into 2 ml EP tubes. For each sample, 1200 μl extraction liquid (methanol:chloroform = 3:1) and 5 μl of L‐2‐chlorophenylalanine were added followed by vortex mixing for 30 s. The samples were homogenized with ball mill at 45 Hz for 4 min and sonicated for 5 min in ice‐water bath. The homogenization and sonication cycle was repeated for five times. Then, the samples were centrifuged at 12000 rpm for 15 min at 4°C. Nine hundred fifty microliters of supernatant was transferred to a fresh 2 ml gas chromatography‐mass spectrometry (GC/MS) glass vial and dried in a vacuum concentrator without heating. Twenty microliters of methoxy amination hydrochloride was added into each dried sample and incubated for 30 min at 80°C. Then, 30 μl of BSTFA regent (1% TMCS, v/v) was added into the sample aliquots, incubated for 1.5 h at 70°C. After cooling to the room temperature, 5 μl of FAMEs was added into each sample. All samples were analysed by gas chromatograph system coupled with a Pegasus HT time‐of‐flight mass spectrometer (GC–TOF–MS).

### 2.24 Quantification and statistical analysis

All data are presented as mean ± standard error of the mean (SEM) or standard deviation (SD). The data were analysed using GraphPad Prism 8.0 (GraphPad Software LLC, San Diego, CA, USA). Data between two groups were analysed by unpaired *t*‐test if the data were in the Gaussian distribution and had equal variance, by unpaired *t*‐test with Welch's correction if the data were in the Gaussian distribution but with unequal variance, or by Mann–Whitney *U*‐test if the data were not normally distributed. For multigroup comparisons with the Gaussian distribution, one‐way analysis of variance (ANOVA) with the Bonferroni multiple comparison test was conducted after the confirmation of homogeneity of variance among the groups by Bartlett's test. For multigroup comparisons with the non‐Gaussian distribution, the Kruskal–Wallis test with Dunn's test was used. Significant differences were declared when *P *< 0.05.

## 3 RESULTS

### 3.1 Melatonergic activation suppresses LPS/IFN‐γ‐stimulated macrophage inflammation

**FIGURE 1:**
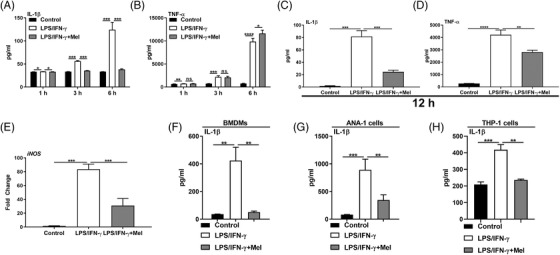
Melatonergic activation inhibits pro‐inflammatory macrophage phenotypes. (A and B) The secretion of interleukin (IL)‐1β (A) and tumour necrosis factor (TNF)‐α (B) from peritoneal exudate macrophages (PEMs) with treatments as indicated (*n* = 3) [lipopolysaccharide (LPS) (1 μg/ml) plus interferon (IFN)‐γ (20 ng/ml) with or without melatonin (1 mM) for 1, 3, 6 h]. (C and D) The secretion of IL‐1β (*n* = 4) (C) and TNF‐α (*n* = 3) (D) from PEMs with treatments as indicated. Results represent three independent experiments. (E) Relative mRNA expression of iNOS in PEMs (*n* = 4). Results represent two independent experiments. Data shown as means ± SEM. (F–H) The secretion of IL‐1β from bone marrow‐derived macrophage (BMDMs) (*n* = 3) (F), ANA‐1 cells (*n* = 4) (G), and THP‐1 cells (*n* = 4) (H) with treatments as indicated. Data were analysed with one‐way ANOVA with Bonferroni correction (A–H) and represented as means ± SD unless indicated. *P < 0.05, **P < 0.01, ***P < 0.001, ****P < 0.0001

Macrophages produce a large amount of pro‐inflammatory cytokines (e.g., IL‐1β) in the context of inflammatory stimuli, such as LPS (plus IFN‐γ).^31^ Increasing evidence shows that melatonin affects the macrophage function^21^; however, it is poorly understood the systematic and delicate mechanism whereby melatonin or melatonergic activation regulates macrophage functions, including the production of inflammatory cytokines. We found that melatonergic signalling activated by melatonin did not influence proliferation and mildly reduced the apoptosis of LPS/IFN‐γ‐activated PEMs (Figure S1A–C). Interestingly, the melatonin treatment merely reduced IL‐1β production in LPS/IFN‐γ‐stimulated PEMs during the early phase of inflammation (Figure 1A,B). However, melatonin highly lowered IL‐1β and tumour necrosis factor (TNF)‐α production and inducible nitric oxide synthase (iNOS) expression at 12 h post LPS/IFN‐γ stimulation (Figure 1C–E). To avoid cellular heterogeneity (especially for conventional PEMs), we then used BMDMs, ANA‐1 cells, and human THP‐1 cells to clarify the effect of melatonergic activation. Melatonin significantly reduced IL‐1β production in BMDMs, ANA‐1 cells, and THP‐1 cells upon prolonged stimulation of pro‐inflammatory signals (24 h for BMDMs and THP‐1 cells; 12 h for ANA‐1 cells) (Figure 1F–H).

**FIGURE 2:**
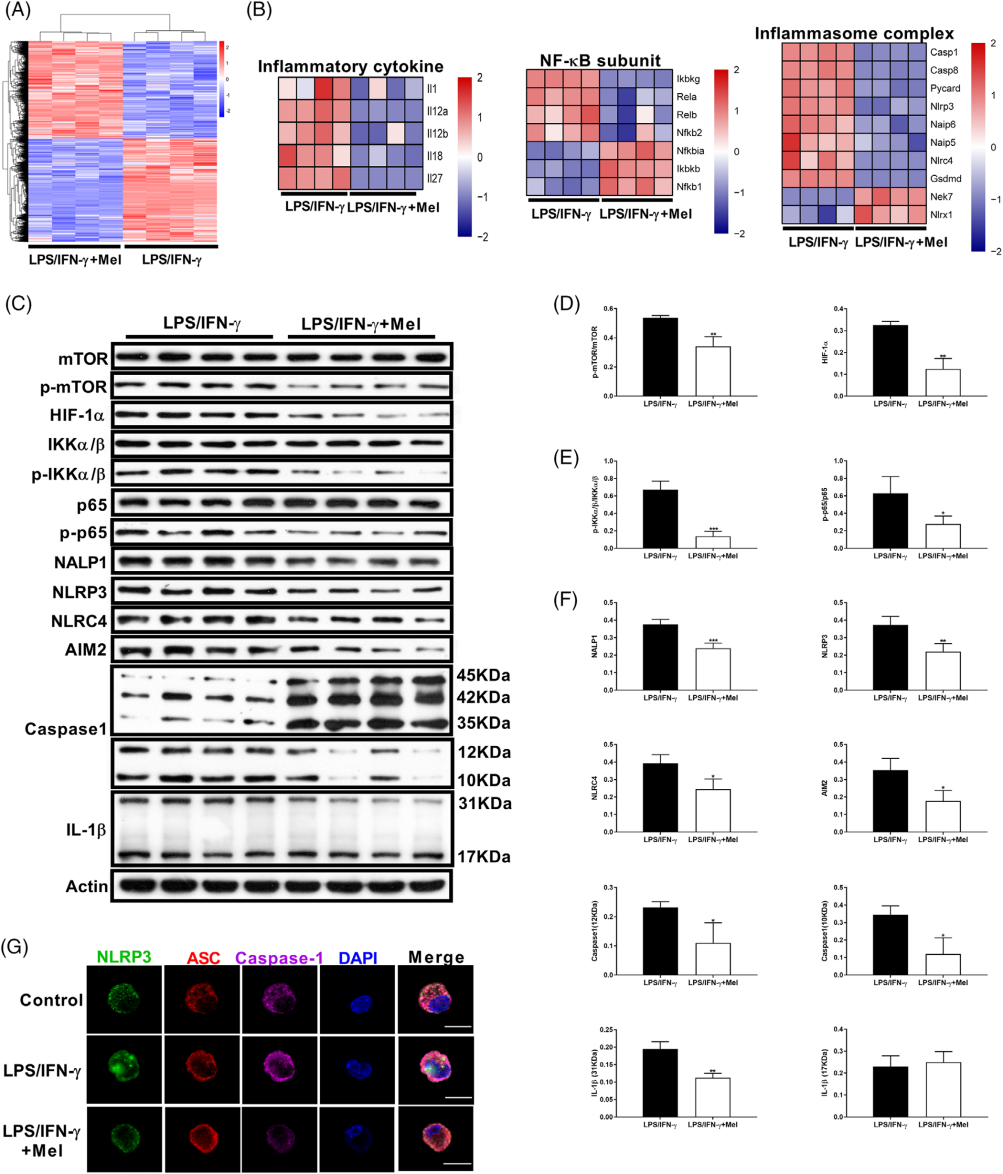
Melatonergic activation alters transcriptomic profile and blocks the activation of inflammatory signalling in lipopolysaccharide (LPS)/IFN‐γ‐stimulated macrophages. (A) Heatmap analysis of up‐regulated (red) or down‐regulated genes (blue) in peritoneal exudate macrophages (PEMs) (*n* = 4). (B) Heatmap analysis of up‐regulated (red) or down‐regulated genes (blue) related to inflammatory cytokine, NF‐κB, and inflammasome complex in PEMs (*n* = 4). (C–F) Protein abundance of mTOR, p‐mTOR, HIF‐1α, and p‐HIF‐1α (D), IKKα/β, p‐IKKα/β, p65, and p‐p65 (E), NALP1, NLRP3, NLRC4, AIM2, Caspase‐1, and interleukin (IL)‐1β (F) in PEMs (*n* = 4). (G) Confocal microscopy of PEMs immunostained for NLRP3 (green), ASC (red) and Caspase‐1 (purple) (*n* = 3). Scale bars, 10 μm. Data were analysed with unpaired *t*‐test (D–F) and represented as means ± SD. *P < 0.05, **P < 0.01, ***P < 0.001

To explore the nature of melatonergic activation‐mediated the suppression of macrophage inflammation, we conducted RNA‐seq analysis on LPS/IFN‐γ‐activated PEMs with or without melatonin treatment. Cluster analysis showed that melatonin highly altered the transcriptomic profile of activated PEMs (Figure 2A). Functional enrichment analysis revealed that the DEGs were mostly enriched in the biological process, including “innate immune response” (Figure S1D). The KEGG pathway analysis suggested that the DEGs were enriched in signalling pathways, including “NF‐κB signalling pathway”, “Toll‐like receptor signalling pathway,” and “RIG‐I‐like receptor signalling pathway” (Figure S1E). Besides, RNA‐seq data showed that melatonin substantially inhibited the expression of pro‐inflammatory polarization‐related cytokines, NF‐κB subunits, and inflammasome complex (Figure 1B). These findings suggest that melatonergic activation transcriptionally suppresses macrophage pro‐inflammatory responses. Notably, our western blotting results validated the data of RNA‐seq, and showed that melatonin blocked the activation of mammalian target of rapamycin (mTOR), hypoxia‐inducible factor (HIF)‐1α, and inflammasome (Figure 2C–F), which are vital for IL‐1β production in pro‐inflammatory macrophages.^34^, ^35^ Interestingly, melatonin reduced the active caspase‐1 p10 release and mature IL‐1β p17 secretion although it had little effect on the abundance of gasdermin D (GSDMD) (Figure S1F), which are accepted hallmarks of inflammasome activation.^36^ Moreover, melatonin blocked NLRP3/ASC/caspase‐1 complex formation in LPS/IFN‐γ‐activated macrophages (Figure 2G).

Subsequently, we sought to ask whether melatonergic activation influences the activation of IL‐4‐activated macrophages. Melatonin did not affect IL‐10 production (Figure S1G); however, it significantly activated phosphorylation of signal transducer and activator of transcription (STAT) 6 (which is associated with anti‐inflammatory macrophage activation^37^) (Figures S1H‐I). Therefore, whether and how melatonergic activation shapes IL‐4‐stimulated macrophage activation is still an open question. Collectively, melatonergic signalling activated by melatonin transcriptionally reduces macrophage pro‐inflammatory responses, especially for lowering IL‐1β production.

### 3.2 Melatonergic activation enhances mitochondrial functions and shifts intracellular metabolism in LPS/IFN‐γ‐stimulated macrophages

**FIGURE 3:**
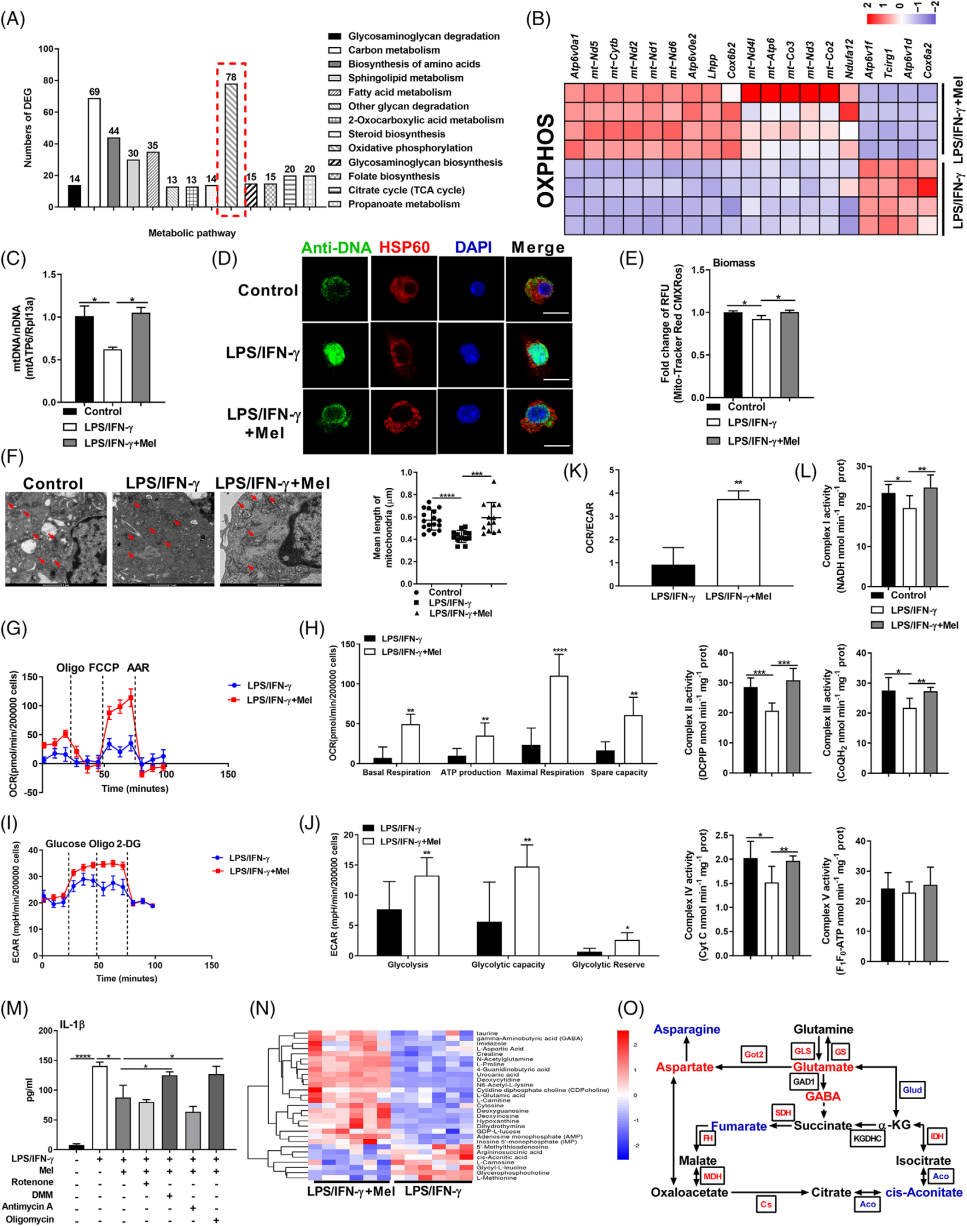
Melatonergic activation enhances mitochondrial functions and shifts intracellular metabolism in lipopolysaccharide (LPS)/ interferon (IFN)‐γ‐stimulated macrophages. (A and B) Histogram of differently expressed genes (DEGs) enriched in the metabolic pathway (A) and heatmap analysis of DEGs enriched in OXPHOS (B) in peritoneal exudate macrophages (PEMs) with treatments as indicated (*n* = 4). (C) Level of mtDNA/nDNA in PEMs (*n* = 3). Results represent two independent experiments. Data shown as means ± SEM. (D) Confocal microscopy of PEMs immunostained for DNA (anti‐DNA, green) and mitochondria (anti‐HSP60, red) (*n* = 3). Scale bars, 10 μm. (E) Fold change of relative fluorescence units (RFU) for Mito‐Tracker Red CMXRos in PEMs (*n* = 3). Results represent two independent experiments. Data shown as means ± SEM. (F) Representative electron photomicrograph of mitochondria (left). Mean length of mitochondria in PEMs (right) (*n* = 13–17). (G–J) The basal respiration, ATP production, maximal respiration, and spare capacity (G and H) or the glycolysis, glycolytic capacity, and glycolytic Reserve (I and J) of PEMs with treatments as indicated (*n* = 10). Results represent two independent experiments. (K) The ratio of basal respiration and glycolysis (*n* = 10). (L) Complex activity (Ⅰ, Ⅱ, III, IV, and V) of PEMs (*n* = 7). Results represent two independent experiments. (M) The secretion of interleukin (IL)‐1β from PEMs with treatments as indicated [in some groups, rotenone (100 nM), dimethyl malonate (DMM) (1 mM), antimycin A (1 μM), and oligomycin (1 μM) was respectively added with melatonin] (*n* = 3–4). Results represent three independent experiments. (N) Heatmap analysis of different metabolites in PEMs (*n* = 6). (O) Schematic diagram of selected metabolites involving metabolic pathway. Data were analysed with unpaired *t*‐test (H, J, and K) or one‐way ANOVA with Bonferroni correction (C, E, F, right panel, L, and M) and represented as means ± SD unless indicated. *P < 0.05, **P < 0.01, ***P < 0.001, ****P < 0.0001

Considering the cellular metabolism is responsible for the functional output of immune cells (e.g., macrophages^38^ and T cells^39^), we further analysed the DEGs ascribed to the metabolic pathways obtained from our RNA‐seq analysis. Interestingly, these DEGs were mostly enriched in oxidative phosphorylation (OXPHOS, Figure 3A). Cluster analysis revealed that the most DEGs enriched in OXPHOS were mitochondrial genes (Figure 3B), which suggest that functional alterations in mitochondria are associated with melatonergic activation‐mediated the suppression of macrophage inflammation. Notably, melatonin rescued mtDNA depletion and shaped mtDNA packaging, organization, and distribution (Figure 3C,D), and enhanced biological functions of mitochondria (as evidenced by the altered levels of MMP, ATP, and mtROS) in LPS/IFN‐γ‐activated macrophages (Figure 3E and S2A‐D). It is mentionable that pro‐inflammatory macrophages produce ROS, which inhibits the mitochondrial function and in turn contributes to pro‐inflammatory macrophage polarization.^40^, ^41^ To assess this possibility, we pre‐treated macrophages with the ROS scavenger *N*‐acetylcysteine (NAC) followed by LPS/IFN‐γ treatment with or without melatonin. We found that NAC pretreatment highly reduced pro‐inflammatory macrophage inflammation, and more interestingly, NAC further enhanced the effect of melatonin on IL‐1β production (Figure S2E).

The functional dynamics of metabolism is closely intertwined with alterations in mitochondrial morphology that dictates pro‐inflammatory macrophage activation.^21^, ^42^ Melatonin‐altered mitochondrial dynamics (especially increasing fusion‐related OPA1 expression) and increased the mean length of mitochondria in macrophages (Figures 3F and S2F,G). Subsequently, we determined cellular bioenergetics with seahorse analysis and confirmed that melatonin switched cellular metabolism towards OXPHOS in pro‐inflammatory macrophages (Figure 3G–K). Meanwhile, melatonin enhanced electron transport chain (ETC) activity of LPS/IFN‐γ‐activated PEMs (Figure 3L). To explore the roles of the mitochondrial function in melatonin‐mediated inhibition on IL‐1β production, we used different inhibitors to inhibit the ETC activity. Dimethyl malonate (DMM, specific inhibitor for Complex Ⅱ) and oligomycin (specific inhibitor for Complex V) treatment significantly rescued the IL‐1β production from melatonin‐treated macrophages (Figure 3M). These convincing results suggest that melatonergic activation enhances mitochondrial functions of LPS/IFN‐γ‐stimulated macrophages.

Pro‐inflammatory macrophages rely mainly on glycolysis and present two breaks on the tricarboxylic acid (TCA) cycle.^43^ Intriguingly, our RNA‐seq data showed that melatonin could “repair” the broken TCA cycle flux in pro‐inflammatory macrophages (Figure S2H,I). Next, we performed metabonomics analysis to fully understand the alterations in cellular metabolism of LPS/IFN‐γ‐activated macrophages in the context of the melatonin treatment. Our data from 200MRM method (targeted metabonomics)^44^ revealed that melatonin substantially reprogrammed the intracellular metabolism in pro‐inflammatory macrophages ( Figure 3N and S2J). As melatonin is not included in the database of the 200MRM method, the list of different metabolites is lack of melatonin (Figure 3N). We then analysed the metabolites with the untargeted GC–TOF–MS analysis (Figure S2K,L), and found that melatonin was accumulated in macrophages upon the melatonin treatment (Figure S2L). Seemingly, melatonergic signalling activated by melatonin generated a complete TCA cycle in pro‐inflammatory macrophages via providing the intermediate metabolites (e.g., GABA) and increasing the expression of related enzymes (mainly SDH and IDH) (Figures 3O and S2M). Altogether, melatonergic activation reduces the production of IL‐1β in LPS/IFN‐γ‐activated macrophages, which is associated with enhancing mitochondrial functions and reprogramming intracellular metabolism.

### 3.3 Melatonergic activation mediates inhibition of IRF7 to lower IL‐1β production

**FIGURE 4:**
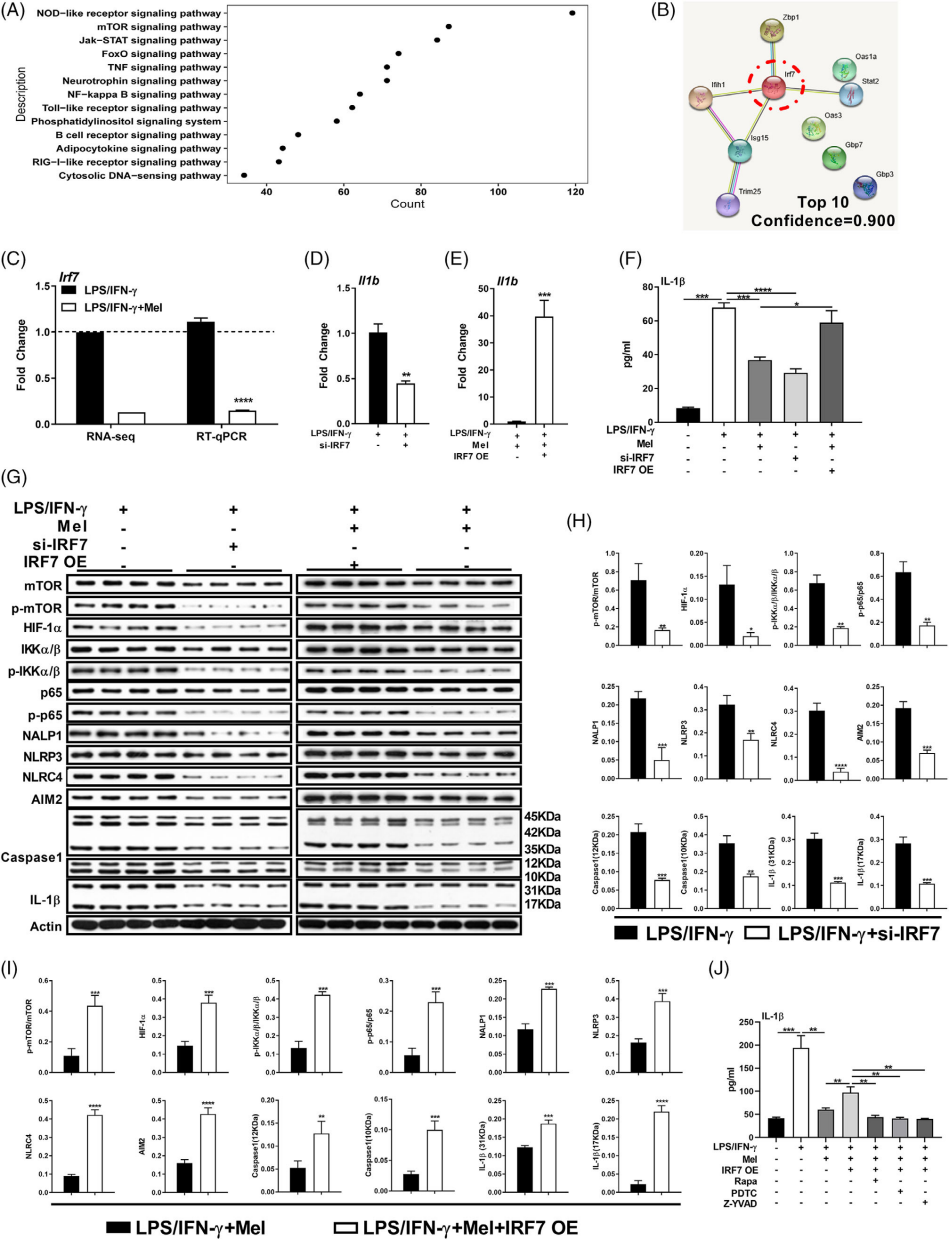
Melatonergic activation mediates inhibition of interferon regulatory factor 7 (IRF7) to lower interleukin (IL)‐1β production. (A) Scatter diagram of differently expressed genes (DEGs) enriched in cellular pathway in lipopolysaccharide (LPS)/ interferon (IFN)‐γ‐stimulated peritoneal exudate macrophages (PEMs) with or without melatonin treatment (1 mM) (*n* = 4). (B) STRING analysis of the selected DEGs. (C) Relative mRNA expression of interferon regulatory factor 7 (IRF7) in PEMs with treatments as indicated based on RNA‐Seq analysis and RT‐qPCR method (*n* = 4). Data shown as means ± SEM. (D and E) Relative mRNA expression of IL‐1β in PEMs with treatments as indicated (si‐IRF7: IRF7 silencing; IRF7 OE: IRF7 overexpression, the same as below unless indicated) (*n* = 3–4). Results represent three independent experiments. Data shown as means ± SEM. (F) The secretion of IL‐1β from PEMs with treatments as indicated (*n* = 3–4). Results represent three independent experiments. (G–I) Protein abundance of mTOR, p‐mTOR, HIF‐1α, p‐HIF‐1α, IKKα/β, p‐IKKα/β, p65, p‐p65, NALP1, NLRP3, NLRC4, AIM2, Caspase‐1, and IL‐1β in PEMs with treatments as indicated (*n* = 4). (J) The secretion of IL‐1β from PEMs (in some groups, pre‐treated with Rapamycin: 5 μM, PDTC: 5 μM, or Z‐YVAD: 10 μM, for 1 h) (*n* = 3). Results represent four independent experiments. Data were analysed with unpaired *t*‐test (C, D, E, H, and I) or one‐way ANOVA with Bonferroni correction (F and J) and represented as means ± SD unless indicated. *P < 0.05, **P < 0.01, ***P < 0.001, ****P < 0.0001

To explore the intrinsic mechanism by which melatonergic activation inhibits pro‐inflammatory macrophage inflammation, especially for IL‐1β production, we further analysed the DEGs enriched in the cellular signalling that are relevant to macrophage pro‐inflammatory responses obtained from RNA‐seq analysis (Figure 4A). Intriguingly, interferon regulatory factor 7 (IRF7) was taken as the hub gene involving inflammation‐associated signalling pathways by Connectivity and STRING analysis (Figures 4B and S3A‐B). Of note, the melatonin treatment substantially reduced *Irf7* expression (Figure 4C), indicating that *Irf7* might be positively correlated with macrophage inflammation. Subsequently, we genetically modulated the IRF7 to validate the crucial role of IRF7 in guiding macrophage inflammation. IRF7 silencing inhibited IL‐1β expression and production in pro‐inflammatory macrophages; while IRF7 overexpression rescued IL‐1β expression and production in pro‐inflammatory macrophages upon melatonergic activation (Figures 4D–F and S3C–G). We also demonstrated that IRF7 overexpression alone further enhanced the production of IL‐1β in LPS/IFN‐γ‐activated macrophages (Figure S3H). These convincing results highlight the critical role of melatonin in regulating IRF7 expression to reduce the IL‐1β production in pro‐inflammatory macrophages. Conformably, IRF7 silencing blocked the activation of signalling pathways which are associated with pro‐inflammatory macrophage activation and reduced protein abundance of IL‐1β in LPS/IFN‐γ‐stimulated macrophages (Figure 4G,H). Notably, IRF7 overexpression counteracted the suppressive effects of melatonin on pro‐inflammatory signalling pathways and IL‐1β level in macrophages (Figure 4G and I). To further validate the aforementioned processes, we used Rapamycin, pyrrolidine dithiocarbamate (PDTC), and Z‐YVAD to block the activation of mTOR, p65, and Caspase‐1, respectively. These inhibitors all significantly reduced IL‐1β production in melatonin‐treated pro‐inflammatory macrophages with IRF7 overexpression (Figure 4J). These interesting data confirmedly suggest that IRF7 participates in the regulation of pro‐inflammatory macrophage inflammation. We also wanted to know whether IRF7 is also responsible for intracellular metabolism (Figure S2M); nevertheless, the expression of metabolism‐related genes was independent of IRF7 (Figure S3I). Taken together, melatonergic signalling activated by melatonin reduces the IL‐1β production may be through blocking the activation of inflammatory signalling by inhibiting IRF7 expression.

### 3.4 IFNGR2‐JAK1/2‐STAT1‐IRF7 signalling contributes to the reduced IL‐1β production in macrophages upon melatonergic activation

**FIGURE 5:**
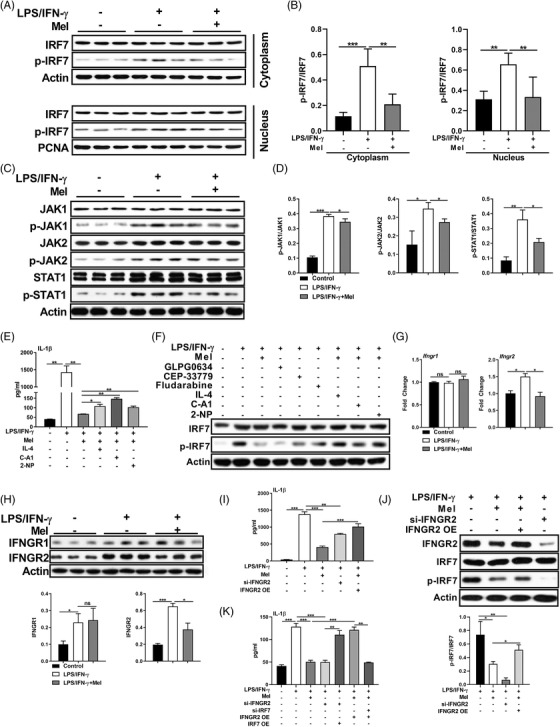
IFNGR2‐JAK1/2‐STAT1‐IRF7 signalling contributes to the reduced interleukin (IL)‐1β production in macrophages upon melatonergic activation. (A and B) Protein abundance of interferon regulatory factor 7 (IRF7) and p‐IRF7 in cytoplasm and nucleus of peritoneal exudate macrophages (PEMs) with treatments as indicated (*n* = 3). Results represent three independent experiments. (C and D) Protein abundance of JAK1, p‐JAK1, JAK2, p‐JAK2, STAT1, and p‐STAT1 in PEMs (*n* = 3). Results represent two independent experiments. (E) The secretion of IL‐1β from PEMs with treatments as indicated (in some groups, pre‐treated with IL‐4: 10 ng/ml, C‐A1: 10 μM, 2‐NP: 45 μM, for 1 h, the same as below unless indicated) (*n* = 3). Results represent three independent experiments. (F) Protein abundance of IRF7 and p‐IRF7 in PEMs with treatments as indicated (in some groups, pre‐treated with GLPG0634: 10 μM, CEP‐33779: 1 μM, Fludarabine: 10 μM, for 1 h) (*n* = 3). Results represent three independent experiments. (G) Relative mRNA expression of IFNGR1 and IFNGR2 in PEMs (*n* = 3). Results represent three independent experiments. Data shown as means ± SEM. (H) Protein abundance of IFNGR1 and IFNGR2 in PEMs (*n* = 3). Results represent two independent experiments. (I and J) The secretion of IL‐1β (I) and protein abundance of IRF7 and p‐IRF7 (J) in PEMs with treatments as indicated (si‐IFNGR2: IFNGR2 silencing; IFNGR2 OE: IFNGR2 overexpression, the same as below unless indicated) (*n* = 3). (K) The secretion of IL‐1β from PEMs with treatments as indicated (*n* = 3). Results represent three independent experiments. Data were analysed with one‐way ANOVA with Bonferroni correction (B, D, E, G, H, I, J, and K) and represented as means ± SD unless indicated. *P < 0.05, **P < 0.01, ***P < 0.001

Subsequently, we explored how melatonin affects IRF7 expression. Given that IRF7 could be a transcription factor (TF),^45^ thus, we first envisioned that whether melatonin alters its nuclear translocation. Here, we found that melatonin reduced IRF7 abundance in both cytoplasm and nucleus (Figure 5A,B). Then, we wanted to know whether some regulators [e.g., myeloid differentiation primary response protein 88 (MyD88), TNF receptor associated factor (TRAF) 6, and TRAF3] contribute to IRF7 expression and activation.^46^ Our results demonstrated that LPS‐mediated toll‐like receptor (TLR4)‐MyD88‐TRAF6 or TLR4‐Toll/IL‐1R domain‐containing adaptor‐inducing IFN‐β (TRIF)‐TRAF3 signalling was not associated with the inhibition of the IRF7 expression in macrophages caused by the melatonin treatment (Figure S4A–D). Notably, melatonin reduced IRF7 activation in LPS/IFN‐γ‐activated macrophages even during the early phase of inflammation (at 3 h and 6 h post stimulation) (Figure S4A,B), highlights the critical role of IRF7 in directing macrophage inflammation. These aforementioned findings indicate that there exists other signalling involving IRF7 expression and IL‐1β production in macrophages upon melatonergic activation.

We found that melatonin significantly inactivated IFN‐γ‐mediated JAK1/2‐STAT1 (Figure 5C,D), which is related to IRF7 expression.^47^, ^48^ Notably, melatonin increased the protein expression of suppressor of cytokine signalling (SOCS) 1 (which negatively regulates JAK1/2‐STAT1 activation^49^, ^50^) (Figure S4E). To confirm the role of JAK1/2‐STAT1 in mediating macrophage IL‐1β production, we used GLPG0634, CEP‐33779, and Fludarabine to separately inactivate JAK1, JAK2, and STAT1 in LPS/IFN‐γ‐stimulated macrophages. Inhibition of JAK1, JAK2, or STAT1 lowered IL‐1β production and IRF7 activation in pro‐inflammatory macrophages (Figures 5F and S4F). Then, we applied IL‐4, C‐A1, and 2‐NP to separately activate JAK1, JAK2, and STAT1 in LPS/IFN‐γ‐stimulated macrophages upon melatonergic activation. Interestingly, activation of JAK1, JAK2, or STAT1 highly rescued IL‐1β production and IRF7 activation in LPS/IFN‐γ‐stimulated macrophages upon melatonergic activation (Figures 5E,F). Thus, these results confirm that melatonergic activation reduces IL‐1β production in LPS/IFN‐γ‐activated macrophages by inhibiting IRF7 expression via blocking JAK1/2‐STAT1 activation.

We are particularly interested in seeking the upstream signalling of JAK1/2‐STAT1‐IRF7 pathway. IFN‐γ mediates JAK‐STAT signalling through IFNGR^51^; noteworthy, IFN‐γ signalling is impaired in melatonin‐treated pro‐inflammatory macrophages, as evidenced by selectively lower mRNA expression and protein level of IFNGR2 (Figure 5G,H). Moreover, IFNGR2 silencing inhibited IL‐1β production and IRF7 activation in LPS/IFN‐γ‐stimulated macrophages; while IFNGR2 overexpression rescued IL‐1β production as well as IRF7 activation in pro‐inflammatory macrophages treated with melatonin (Figures 5I,J and S4G), suggesting that IFNGR2 functions as the upstream signalling of the JAK1/2‐STAT1‐IRF7 pathway. More importantly, IRF7 overexpression rescued IL‐1β production in pro‐inflammatory macrophages transfected with si‐IFNGR2, while IRF7 silencing reduced L‐1β production in IFNGR2‐overexpressing pro‐inflammatory macrophages with melatonin treatment (Figure 5K). Collectively, melatonergic activation reduces IL‐1β production in LPS/IFN‐γ‐stimulated macrophages via IFNGR2‐JAK1/2‐STAT1‐IRF7 signalling.

### 3.5 Melatonergic activation transcriptionally inhibits IFNGR2 expression to reduce IL‐1β production

**FIGURE 6:**
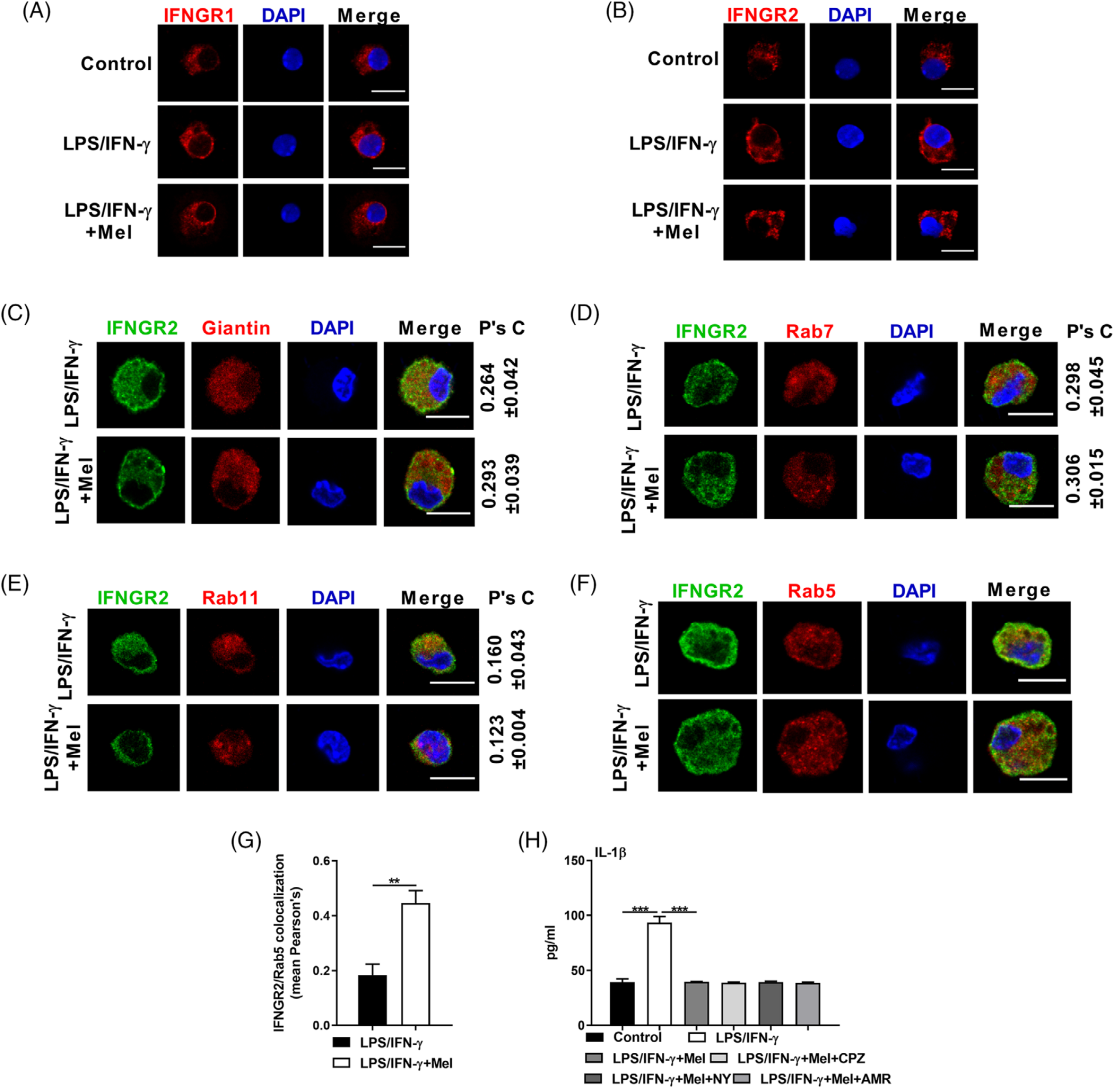
Melatonergic activation affects the distribution of IFNGR2 in lipopolysaccharide (LPS)/interferon (IFN)‐γ‐stimulated macrophages. (A and B) Confocal microscopy of peritoneal exudate macrophages (PEMs) immunostained for IFNGR1 (red) (A) and IFNGR2 (red) (B) with treatments as indicated (*n* = 3). Scale bars, 10 μm. (C–F) Confocal microscopy of PEMs immunostained for IFNGR2 (green) and Giantin (red) (21–28 cells) (C), Rab7 (red) (38–43 cells) (D), Rab11 (red) (23–29 cells) (E), and Rab5 (red) (17–38 cells). Scale bars, 10 μm. (G) Histogram of IFNGR2/Rab5 colocalization. (H) The secretion of interleukin (IL)‐1β from PEMs with treatments as indicated (in some groups, CPA: 10 μM, NY: 50 μg/ml, AMR: 50 μM, was respectively added with melatonin) (*n* = 3). Results represent three independent experiments. Data shown as means ± SD. Data were analysed with unpaired *t*‐test (C–G) or one‐way ANOVA with Bonferroni correction (H) and represented as means ± SEM unless indicated. **P < 0.01, ***P < 0.001

The distribution of IFNGR2 also highly affects the activation of JAK1/2‐STAT1 signalling.^52^ Intriguingly, IF analysis showed that melatonin selectively decreased IFNGR2 on the membrane and increased IFNGR2 in the cytoplasm of LPS/IFN‐γ‐activated macrophages (Figure 6A,B). The number of receptors on the membrane is decided by a series of dynamic processes (i.e., delivery of *de novo* synthesis, endocytosis, recycling, and degradation).^52^ We set out to clarify which part of the processes was affected in LPS/IFN‐γ‐stimulated macrophages upon melatonergic activation. Our IF analysis reported the comparable colocalization between IFNGR2 and Giantin (Golgi marker, represents *de novo* synthesis), Rab7 (lysosome marker, represents degradation), and Rab11 (recycling) in pro‐inflammatory macrophages with melatonin treatment (Figures 6C–E), while the increased colocalization between IFNGR2 and Rab5 (endocytosis) was observed in LPS/IFN‐γ‐activated macrophages after the melatonin treatment (Figure 6F,G). However, endocytosis inhibitors [including chlorpromazine (CPZ), nystatin (NY), and amiloride (AMR)] failed to rescue IL‐1β production in macrophages treated with melatonin (Figure 6H), indicating that the increased endocytosis of IFNGR2 may contribute to, but is not sufficient for melatonergic activation‐mediated inhibition of macrophage pro‐inflammatory responses.

**FIGURE 7:**
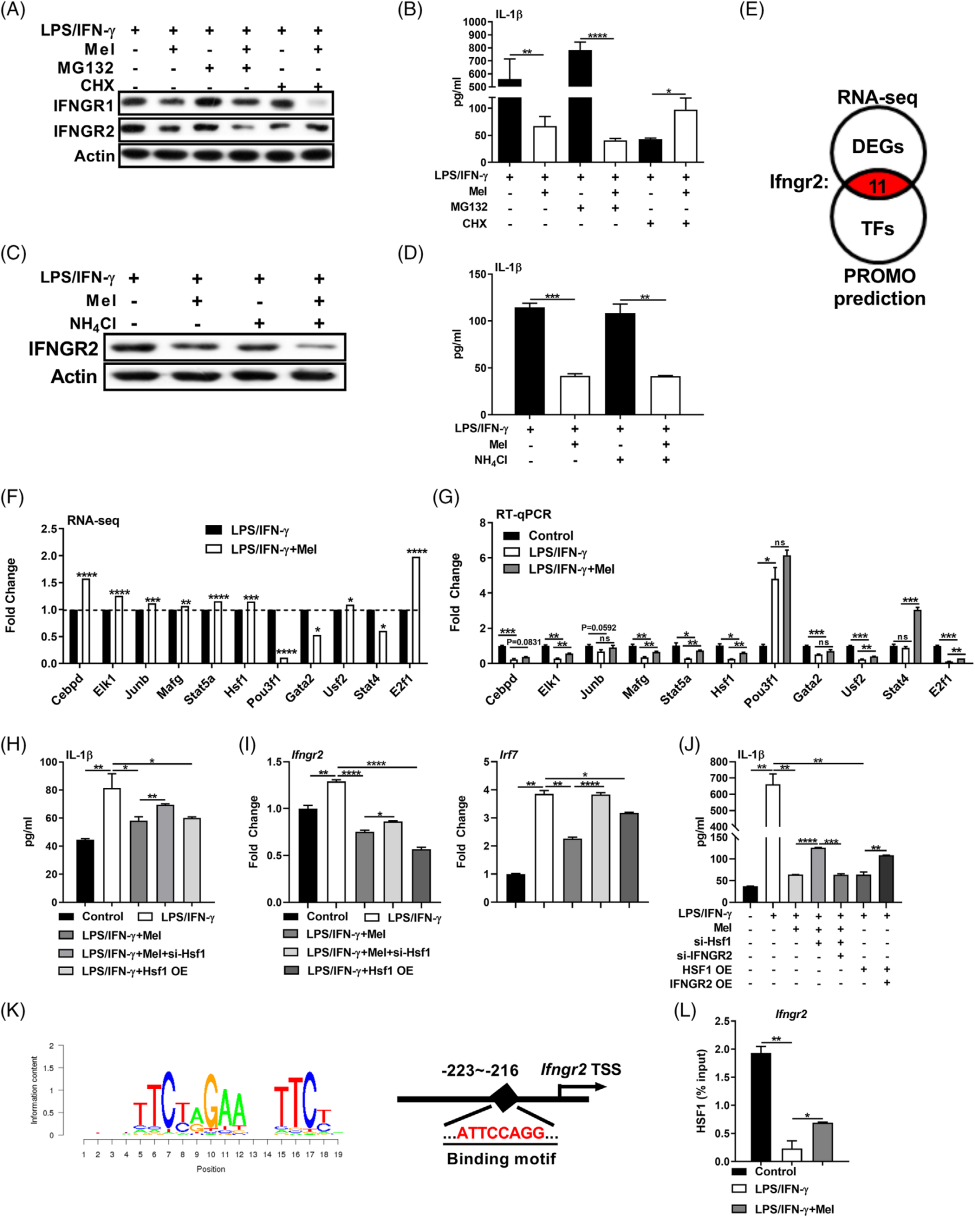
Melatonergic activation transcriptionally inhibits IFNGR2 expression to reduce interleukin (IL)‐1β production. (A) Protein abundance of IFNGR1 and IFNGR2 in peritoneal exudate macrophages (PEMs) with treatments as indicated [in some groups, CHX (10 μM), or MG132 (10 μM) was respectively added with melatonin] (*n* = 3). (B) The secretion of IL‐1β from PEMs with treatments as indicated in (A) (*n* = 3). Data shown as means ± SD. (C) Protein abundance of IFNGR2 in PEMs with treatments as indicated (in some group, NH_4_Cl: 10 mM was added with melatonin) (*n* = 3). (D) The secretion of IL‐1β from PEMs with treatments as indicated in (C) (*n* = 3). Data shown as means ± SD. (E) Schematic diagram of transcription factors (TFs) accounted for the regulation of IFNGR2. (F and G) Relative mRNA expression of selected TFs in PEMs based on RNA‐seq analysis (*n* = 4) (F) and RT‐qPCR method (*n* = 3) (G). (H) The secretion of IL‐1β from PEMs with treatments as indicated (si‐Hsf1: Hsf1 silencing; Hsf1 OE: Hsf1 overexpression, the same as below unless indicated) (*n* = 3). Results represent three independent experiments. Data shown as means ± SD. (I) Relative mRNA expression of IFNGR2 (left) and interferon regulatory factor 7 (IRF7) (right) in PEMs with treatments as indicated in (H) (*n* = 3). Results represent three independent experiments. (J) The secretion of IL‐1β from PEMs with treatments as indicated (*n* = 3). Results represent three independent experiments. Data shown as means ± SD. (K) Binding motif analysis of *Ifngr2* transcriptional start site (TSS) by JASPAR. (L) CHIP‐qPCR analysis of HSF1 enrichment at the promoter of the *Ifngr2* gene (*n* = 3). Data were analysed with unpaired *t*‐test (F) or one‐way ANOVA with Bonferroni correction (B, D, G, H, I, J, and L) and represented as means ± SEM unless indicated. *P < 0.05, **P < 0.01, ***P < 0.001, ****P < 0.0001

Then, we investigated whether the reduced expression of IFNGR2 protein in melatonin‐treated pro‐inflammatory macrophages could be due to aberrant protein translation and/or degradation. Interestingly, the translation inhibitor cycloheximide (CHX) significantly lowered IFNGR2 protein amount and IL‐1β production in LPS/IFN‐γ‐stimulated macrophages (Figure 7A,B). More importantly, CHX treatment blocked the effect of melatonin on the expression of IFNGR2 and IL‐1β production in pro‐inflammatory macrophages (Figure 7A,B). By contrast, inhibiting either proteasome‐mediated protein degradation with MG132 or lysosome‐mediated protein degradation with NH_4_Cl, failed to block the influence of melatonin on IFNGR2 level and IL‐1β production in pro‐inflammatory macrophages (Figure 7A–D). Thus, it seems that translation inhibition is the main reason for IFNGR2 impairment in LPS/IFN‐γ‐stimulated macrophages upon melatonergic activation. Notably, our RNA‐seq data showed that most of DEGs related to translational initiation factors, elongation factors, and termination factor were down‐regulated, while *Pdcd4* (translational repressor) was up‐regulated in macrophages upon melatonergic activation (Figure S5A), implying that melatonin induces the inhibition of protein translation in pro‐inflammatory macrophages.

Given that melatonin suppressed IFNGR2 expression in LPS/IFN‐γ‐stimulated macrophages both at mRNA and protein level (Figure 5G,H), we further focused on the transcriptional regulation of IFNGR2. Combined with the RNA‐seq data, we identified 11 TFs that could occupy at the *Ifngr2* gene promoter region using PROMO (http://alggen.lsi.upc.es, Figure 7E,F). Our RT‐qPCR results revealed that Elk1, Mafg, Stat5a, Hsf1, Usf2, and E2f1 might participate into modulating *Ifngr2* transcription (Figure 7G). Elk1, Hsf1, and E2f1 were of the upmost interest for the further investigation as they could be affected by melatonin.^53^, ^54^, ^55^ Of note, we found only Hsf1 silencing or overexpression affected the mRNA expression of IFNGR2 and IRF7 as well as IL‐1β production in pro‐inflammatory macrophages (Figures 7H,I and S5B–D). As the melatonin treatment promoted the expression of Hsf1 in pro‐inflammatory macrophages, thus, we silenced Hsf1 in melatonin‐treated pro‐inflammatory macrophages, while overexpressed Hsf1 in pro‐inflammatory macrophages. Hsf1 silencing rescued the IL‐1β production in melatonin‐treated pro‐inflammatory macrophages, while Hsf1 overexpression mirrored melatonin‐mediated suppressive effect on IL‐1β production in pro‐inflammatory macrophages (Figure 7H–J). These results indicated that melatonin negatively regulates *Ifngr2* expression through inducing Hsf1 expression in LPS/IFN‐γ‐stimulated macrophages. To validate the aforementioned process, we applied simultaneously interfering expression of Hsf1 and IFNGR2 in melatonin‐treated pro‐inflammatory macrophages, while simultaneously overexpressing of Hsf1 and IFNGR2 in pro‐inflammatory macrophages. Expression of IFNGR2 highly blocked the influence of Hsf1 on the IL‐1β production (Figure 7J), suggesting that IFNGR2 is the downstream of Hsf1. Moreover, we identified the binding site for Hsf1 in the *Ifngr2* gene promoter region based on the JASPAR (http://jaspar.genereg.net) (Figure 7K); unfortunately, we failed to conduct dual‐luciferase reporter assay due to the difficulty in constructing *Ifngr2* luciferase promoter construct for the following reasons: (1) repeated sequence and (2) almost 100% GC content in the *Ifngr2* gene promoter sequence (Figure S5E). Instead, CHIP‐qPCR assay demonstrated that melatonergic activation mediated direct binding of Hsf1 to the identified site in the promoter region of *Ifngr2* (Figure 7L). Summarily, melatonergic activation transcriptionally inhibits IFNGR2 expression through upregulating Hsf1, thereby lowering IL‐1β production in LPS/IFN‐γ‐activated macrophages.

### 3.6 MT1‐GSK3β‐Hsf1 axis mediates the quenching of *Ifngr2* transcription

We then explored the elaborated mechanism whereby melatonin influences Hsf1 expression, *Ifngr2* transcription, and subsequent IL‐1β production. First, we used Luzindole, 4‐P‐PDOT, Prazosin, KN‐93, and SR1001 to separately inhibit MT1/2, MT2, MT3, Calmodulin, and RORα/RZR, which are reported to be interacted with melatonin;^56^ however, no significant change of IL‐1β production in LPS/IFN‐γ‐stimulated macrophages with the melatonin treatment was observed after using these inhibitors (Figure S6A). It has been well documented that melatonin activates Sirt1 to exert its anti‐inflammatory properties.^26^ Likewise, the nicotinamide (a pan SIRTs inhibitor) failed to recue IL‐1β production (Figure S6B). The slight effects of these inhibitors on the IL‐1β production might be due to the high concentration of melatonin used in this study. Thus, we envisioned that a procedure via siRNA may be preferable compared to even higher amounts of antagonists (which may turn out to be toxic) for confronting the effects of high concentration of melatonin.

**FIGURE 8:**
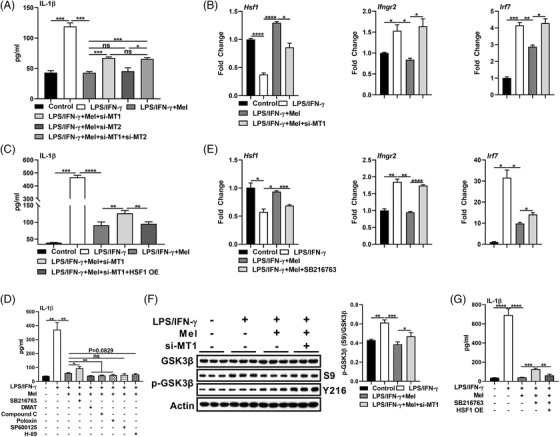
MT1‐GSK3β‐Hsf1 axis mediates melatonin in quenching *Ifngr2* transcription. (A) The secretion of interleukin (IL)‐1β from peritoneal exudate macrophages (PEMs) with treatments as indicated (si‐MT1: MT1 silencing; si‐MT2: MT2 silencing) (*n* = 3). Results represent three independent experiments. (B) Relative mRNA expression of HSF1 (left), IFNGR2 (middle), and IRF7 (right) in PEMs with treatments as indicated (*n* = 3). Results represent three independent experiments. Data shown as means ± SEM. (C) The secretion of IL‐1β from PEMs with treatments as indicated (*n* = 3). Results represent two independent experiments. (D) The secretion of IL‐1β from PEMs with treatments as indicated [in some groups, SB216763 (10 μM), DMAT (10 μM), Compound C (10 μM), Poloxin (10 μM), SP600125 (10 μM), or H‐89(10 μM) was respectively added with melatonin] (*n* = 3). (E) Relative mRNA expression of HSF1 (left), IFNGR2 (middle), and IRF7 (right) in PEMs with treatments as indicated (*n* = 3). Data shown as means ± SEM. (F) Protein abundance of GSK3β and p‐GSK3β (S9 and Y216) in PEMs with treatments as indicated in (B) (*n* = 3). (G) The secretion of IL‐1β from PEMs with treatments as indicated (*n* = 3). Data were analysed with one‐way ANOVA with Bonferroni correction (A‐E, F, right panel, and G) and represented as means ± SD unless indicated. *P < 0.05, **P < 0.01, ***P < 0.001, ****P < 0.0001

Notably, we observed that only MT1 silencing but not MT2 silencing inhibited *Hsf1* mRNA expression, and rescued *Ifngr2* and *Irf7* transcripts and IL‐1β production (Figures 8A,B and S6C,D). Moreover, Hsf1 overexpression blocked the rescue in IL‐1β production after MT1 silencing in melatonin‐treated pro‐inflammatory macrophages (Figure 8C). Subsequently, we investigated the link between MT1 and Hsf1. Melatonin leads to ERK1/2 activation via MT1;^27^ and ERK1/2 activation is supposed to phosphorylate HSF1 at Ser307.^57^ However, ERK1/2 was excluded based on the results: (1) melatonin failed to activate the ERK1/2 in pro‐inflammatory macrophages; (2) MT1 silencing was unable to inhibit the ERK1/2 in melatonin‐treated pro‐inflammatory macrophages; (3) ERK1/2 activator (TPA) did not inhibit IL‐1β production in pro‐inflammatory macrophages; (4) ERK1/2 inhibitor (SCH772984) was incapable of rescuing IL‐1β production in melatonin‐treated pro‐inflammatory macrophages (Figure S6E,F). Likewise, MEK (the upstream signalling of HSF1) was also ruled out due to the little effect was found on phosphorylated HSF1 (Ser326) in pro‐inflammatory macrophages upon melatonergic activation (Figure S6G). These results suggest that there are other mechanisms connecting to the regulation of Hsf1 by MT1.

Then, we screened the signalling downstream of MT1, which could regulate Hsf1, through using various kinase inhibitors include SB216763, DMAT, Compound C, Poloxin, SP600125, and H‐89 targeting GSK3, casein kinase 2 (CK2), AMP‐activated protein kinase (AMPK), polo‐like kinase 1 (PLK1), c‐Jun N‐terminal kinase (JNK), and protein kinase (PKA), respectively. We concluded that MT1‐GSK3β‐Hsf1 axis mediated the inhibition of *Ifngr2* transcription to reduce IL‐1β production by the following evidence: (1) only SB216763 rescued IL‐1β production in melatonin‐treated pro‐inflammatory macrophages (Figure 8D); (2) inhibiting GSK3 by SB216763 decreased *Hsf1* mRNA expression while increased *Ifngr2* and *Irf7* transcripts in melatonin‐treated pro‐inflammatory macrophages (Figure 8E); (3) melatonin activated GSK3β via MT1 by lowering the phosphorylation of GSK3β at Ser9 (representing inactive GSK3β, Figure 8F) instead at Tyr216 (indicating active GSK3β)^58^ (Figure S6H); (4) Hsf1 overexpression was sufficient for reducing SB216763‐triggered high level of IL‐1β production in LPS/IFN‐γ‐activated macrophages with melatonin treatment (Figure 8G). Collectively, melatonin acts primarily via MT1 to lower the inactive GSK3β to increase *Hsf1* expression, thereby transcriptionally inhibiting IFNGR2 and subsequently reducing macrophage inflammation, especially for IL‐1β production.

### 3.7 Melatonergic activation amplifies host protective responses to *Pasteurella multocida* infection

**FIGURE 9:**
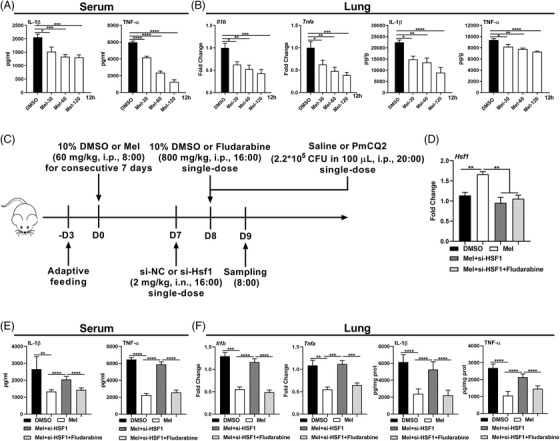
Melatonergic activation amplifies host protective responses to *Pasteurella multocida* infection. (A) Levels of interleukin (IL)‐1β and tumour necrosis factor (TNF)‐α in serum of PmCQ2‐infected mice pre‐treated with or without melatonin (30, 60, or 120 mg/kg BW) at 12 h post infection (*n* = 8). (B) Levels of IL‐1β and TNF‐α in lungs of PmCQ2‐infected mice with the treatments as indicated in (A) (*n* = 6–8). RT‐qPCR data shown as means ± SEM. (C) Schematic diagram for the description of treatments in mice. (D) Relative mRNA expression of HSF1 in lungs of PmCQ2‐infected mice with the treatments as indicated (DMSO: 10% v:v; melatonin: 60 mg/kg BW; si‐HSF1: Hsf1 silencing in vivo; Fludarabine: 800 mg/kg BW in vivo, the same as below) at 12 h post infection (*n* = 5–6). (E) Levels of IL‐1β and TNF‐α in serum of PmCQ2‐infected mice with the treatments as indicated in (D) (*n* = 6). (F) Levels of IL‐1β and TNF‐α in lungs of PmCQ2‐infected mice with the treatments as indicated in (D) (*n* = 6–8). RT‐qPCR data shown as means ± SEM. Data were analysed with unpaired *t*‐test (A and B) or one‐way ANOVA with Bonferroni correction (D–F) and represented as means ± SD unless indicated. *P < 0.05, **P < 0.01, ***P < 0.001, ****P < 0.0001

Our previous study demonstrated that *P. multocida* (PmCQ2) infection induces inflammatory responses in mouse lung, which is largely dependent on macrophages.^31^ To explore whether melatonergic signalling activated by melatonin could enhance host defense against PmCQ2 infection, ICR mice were intraperitoneal (i.p.) injected with different doses of melatonin (30, 60, and 120 mg/kg BW) for consecutive 7 days followed by PmCQ2 infection (i.p.). It should be noted that the natural infectious route for PmCQ2 is through intranasal infection, but we previously found that intranasal infection has a poor reproducibility among mice.^59^ Moreover, there are some similarities between intranasal infection and intraperitoneal infection, including the pathological alterations and expression of inflammatory cytokines.^60^, ^61^ Thus, intraperitoneal infection was used in this study. Considering PmCQ2 causes severe respiratory diseases, therefore, the cytokines in serum and lungs but not peritoneal cavity were assayed. Notably, we found that the levels of IL‐1β and TNF‐α in the serum and lung were substantially reduced at 12 h (Figure 9A,B), and even at 16 h, 24 h, and 32 h post PmCQ2 infection by melatonin injection (Figures S7A–F). It is mentionable that melatonin might have a broader influence on the production of IL‐1β than TNF‐α during the late phase of inflammation (Figure S7A–F).

We then validated the roles of Hsf1 and STAT1 in vivo, ICR mice were injected with 60 mg/kg BW melatonin for consecutive 7 days (i.p.) followed by intranasally (i.n.) treated with 2 mg/kg BW si‐Hsf1 (2OMe+5Chol) at 24 h prior to inhibiting STAT1 in vivo using Fludarabine (800 mg/kg BW, i.p.); the mice were then infected with PmCQ2 (Figure 9C). Hsf1 siRNA successfully inhibited *Hsf1* expression in murine lung (Figure 9D). Of note, we demonstrated that si‐Hsf1 counteracted melatonin‐mediated suppressive effects on the levels of IL‐1β and TNF‐α in the serum and lung at 12 h post PmCQ2 infection (Figure 9E,F). Interestingly, inhibiting STAT1 in vivo could mirror the function of melatonin (Figure 9E,F). Taken together, these results indicate that melatonergic signalling activated by melatonin restrains pro‐inflammatory cytokine production in vivo during PmCQ2 infection.

## 4 DISCUSSION

As an immunotransmitter molecule, melatonin regulates immune responses; nevertheless, the intrinsic mechanism is still not clear. Here, we demonstrate that melatonin causes a transcriptional downregulation of IFNGR2 and impairs canonical signalling events in LPS/IFN‐γ‐stimulated macrophages, thereby reducing IL‐1β production.

Multiple cellular pathways contribute to the activation of macrophages.^62^ In this study, we find that melatonin suppresses the activation of several pathways, including mTOR, NF‐κB, and NLRP3 inflammasome. Other molecular regulators (e.g., c‐MYC) seem to additionally contribute, but these require a broader experimental confirmation. Notably, we reveal that IRF7 participates in mediating suppressive effects of melatonin in modulating the aforementioned inflammatory pathways. NF‐κB is the main TF controlling IL‐1β gene induction (namely pro‐IL‐1β), and caspase‐1 is the major protease required for pro‐IL‐1β processing, leading to mature IL‐1β production and release, especially in macrophages.^63^, ^64^, ^65^ Generally, in most cases, IRF7 and NF‐κB are synchronously expressed in the cells under pro‐inflammatory stimuli; in some special cases, IRF7 inhibits the NF‐κB pathway^66^ or NF‐κB is essential for the induction of IRF7.^67^ However, the mechanisms connecting to the aforementioned processes are still unavailable. In our study, we suggest that IRF7 might positively regulate the NF‐κB pathway; nevertheless, the potential mechanisms remain to be revealed. Furthermore, it is still unclear how IRF7 affects the activation of mTOR and NLRP3 inflammasome.

Numerous signalling cascades (e.g., pathogen recognition receptors) activate IRF7,^46^ which serves as a key regulator in regulating innate and adaptive immunity.^68^ Indeed, upregulation of IRF7 has been found in M1‐like (pro‐inflammatory) macrophages^69^; and in turn, IRF7 favours in regulating monocyte differentiation to macrophages^70^ and controlling M1‐like polarization.^71^ Consistent with the previous study,^72^ here, we also show that melatonin reduces IRF7 expression, thereby inhibiting pro‐inflammatory macrophage inflammation. It should be mentioned that IRF7 could undergo various post‐translational modifications (e.g., phosphorylation, ubiquitination, sumoylation, and acetylation);^46^ thus, it is meaningful to investigate whether melatonin affects modifications of IRF7 in the future.

The mitochondrial functions (including regulation of cellular metabolism) are associated with mitochondrial morphology (controlled by fission and fusion), which is closely interwined with macrophage polarization.^21^, ^31^ Here, we find that melatonin alters mitochondrial morphology, increases fusion (mainly targeting OPA1), and enhances OXPHOS in LPS/IFN‐γ‐stimulated macrophages. These results are conceivable since OPA1 directs mitochondrial morphology and cristae structure in cells,^73^ and cells with OPA1 deficiency have disorganized cristae and reduced OXPHOS.^74^, ^75^ The inconsistent results about other mitochondrial dynamics‐related proteins (especially for DRP1 and MFN1/2) are also observed in our experimental setting; however, the possible reasons remain to be characterized. It has been noted that melatonin shapes intracellular metabolism (e.g., altered levels of aspartate, asparagine, and GABA) of pro‐inflammatory macrophages. Indeed, we illustrate that aspartate, asparagine, and GABA influence IL‐1β production in macrophages with LPS/IFN‐γ stimulation (unpublished data). These findings indicate that melatonin might restrain macrophage inflammation through these TCA cycle intermediates which are traditionally linked to OXPHOS.^76^ Itaconate, which is derived from *cis*‐aconitate intermediates, is identified as an anti‐inflammatory signal.^77^ Although the reduced cellular *cis*‐aconitate level is observed (Figure 3N,O), we fail to find any alteration in levels of cellular itaconate in macrophages treated with melatonin. Notably, the expression of the related enzymes that involve in the production of these TCA cycle intermediates is independent of IRF7, suggesting that alterations of cellular metabolism and cellular pathways are coordinate outcomes triggered by melatonin; nevertheless, how melatonin affect accumulation of these cellular metabolites as well as the distinct mechanism(s) underpinning expression of metabolic enzymes in macrophages remain to be understood.

Complex molecular mechanisms (including transcriptional and post‐transcriptional aspects) connecting IFN‐γ engagement with functional IFNGR to govern JAK/STAT signalling.^78^, ^79^ As mentioned above, diverse signalling cascades are responsible for the activation of IRF7; currently, it is not the canonical signalling (i.e., TLR4‐MyD88‐TRAF6 and TLR4‐TRIF‐TRAF3) but JAK1/2‐STAT1 signalling contributes to melatonin‐mediated IRF7 inactivation, which suggests that IFNGR signalling is defective. The level of functional receptors is dependent of *de novo* synthesis, endocytosis, recycling, and degradation^52^; and functional IFNGR includes IFNGR1 (which is constitutively expressed) and IFNGR2 (which controls signalling upon stimulation).^80^ Correspondingly, we observe that melatonin has little effects on membrane translocation and expression of IFNGR1, but highly affects endocytosis and expression (at both protein and mRNA level) of IFNGR2 on macrophages. It is mentionable that melatonin significantly reduces the level of cellular *N*‐acetyl‐d‐galactosamine (GalNAc) in our study (Figure S2L), which is related to glycosylation.^81^ Considering glycosylation of IFNGR2 has been reported to mediate IFN‐γ signalling,^82^ therefore, it is worth investigating whether melatonin influences glycosylation of IFNGR2 to control the subsequent signalling and IL‐1β production. Even so, we emphasize that melatonin mainly mediates transcriptional inhibition of IFNGR2 to reduce IL‐1β production in LPS/IFN‐γ‐activated macrophages. Nevertheless, we still could not rule out the possibility that the regulation of *Ifngr2* in LPS/IFN‐γ‐treated macrophages by melatonin is due to reduced *Ifngr2* mRNA stability.

The activation of HSF1 reduces IL‐1β through the decrease in macrophage NLRP3 inflammasome activity in alcohol‐associated liver injury.^83^ Notably, we also identify *Hsf1* as the key TF for transcriptionally inhibiting IFNGR2 to relieve macrophage inflammation mediated by melatonin. The phosphorylation of TF is supposed to the activation of TF, and p‐HSF1 (Ser326) represents the transcriptional activation of Hsf1; however, we find that melatonin reduces phosphorylation of HSF1 at Ser326, indicating other phosphorylation sites may involve in melatonin‐induced Hsf1 expression in LPS/IFN‐γ‐activated macrophages. Indeed, many phosphorylation sites (including Ser121, Ser303, Ser307, Ser320, Ser363, Ser419, and Thr142, which have suggested to be phosphorylated by AMPK, GSK3β, ERK1/2, PKA, JNK, PLK1, and CK2, respectively) are responsible for Hsf1 regulation by phosphorylation.^84^, ^85^ Although melatonin acts via MT1 to reduce IL‐1β production, it fails to activate ERK1/2 signalling, which excludes the involvement of Ser307. Fortunately, by using various kinase inhibitors, we clarify GSK3β as the crucial regulator for modulating Hsf1 expression; however, this still needs experimental validation (e.g., mutation of phosphorylation site). Intriguingly, p‐GSK3β (Ser9) restrains the increased level of HSF1^86^; currently, we demonstrate that melatonin acts via MT1 to reduce p‐GSK3β (Ser9) to upregulate Hsf1 expression, thereby transcriptionally inhibiting IFNGR2 and subsequently reducing IL‐1β‐dependent inflammation. Recently, vitamin D receptor (VDR) has been considered as a novel melatonin‐binding nuclear receptor due to the direct interaction between melatonin (1 mM) and VDR.^87^ Therefore, it would be interesting to further explore whether VDR mediates the effects of melatonin in modulating GSK3β‐Hsf1‐IFNGR2 axis in the future.

IL‐1β is pathologically activated in inflammatory conditions and excessive IL‐1β exaggerates the severities of inflammatory diseases. Our findings suggest that melatonergic signalling activated by melatonin substantially alleviates hyperinflammatory responses trigged by PmCQ2 infection‐induced pneumonia. Indeed, melatonin is relatively safe with a low risk of side effects.^88^, ^89^ Clinically, melatonin can be applied as an adjuvant drug to facilitate *H. pylori* eradication in patients with gastroduodenal ulcer by the omeprazole treatment.^90^, ^91^ The pathogenesis of a COVID‐19 respiratory infection is associated with the cytokine storm (e.g., excessive IL‐1β and TNF‐α), and COVID‐19 infection causes an impaired melatonin synthesis in macrophage mitochondria due to lacking of acetyl‐coenzyme A, which is an essential co‐factor/substrate for the rate‐limiting enzyme arylalkylamine *N*‐acetyltransferase (AANAT).^92^ Recently, melatonin has been proposed as an adjuvant treatment for patients with COVID‐19.^93^, ^94^ Conceivably, our findings further provide proof‐of‐concept evidence showing that the melatonergic activation could tackle COVID‐19‐induced cytokine storm.

## 5 CONCLUSION

Collectively, we have elucidated that melatonin reduces IL‐1β‐dependent inflammation. Mechanistically, melatonin mediates transcriptional inhibition of IFNGR2 through MT1‐GSK3β‐Hsf1 axis, thereby suppressing the subsequent pro‐inflammatory signalling cascades in macrophages. Thus, manipulation of melatonergic system could be a strategy to prevent and/or treat inflammatory diseases.

## FUNDING

Guangdong Basic and Applied Basic Research Foundation, Grant Number: 2019B1515210002; National Natural Science Foundation of China, Grant Number: 31922079.

## CONFLICT OF INTEREST

The authors declare that there is no conflict of interest that could be perceived as prejudicing the impartiality of the research reported.

## Supporting information

Supporting information

Supporting information

Supporting information

Supporting information

Supporting information

Supporting information

Supporting information

Supporting information
