# Supplementary material for: Melatonergic signalling instructs transcriptional inhibition of IFNGR2 to lessen interleukin‐1β‐dependent inflammation
Source: Clin Transl Med. 2022 Feb 20;12(2):e716. doi: 10.1002/ctm2.716 (PMC8858632; doi:10.1002/ctm2.716)
Supplement: Supplementary file 1 — Supporting information [file CTM2-12-e716-s006.docx]

Supplementary Materials for

**Melatonergic signaling instructs transcriptional inhibition of IFNGR2 to lessen IL-1β-dependent inflammation**

Yaoyao Xia, Qingzhuo Zhang, Yuyi Ye, Xiaoyan Wu, Fang He, Yuanyi Peng, Yulong Yin, Wenkai Ren

This PDF file includes:

Table S1

Figs. S1 to S7

**Supplementary Materials**

**Table. S1 Key resource table.**

| REAGENT or RESOURCE | SOURCE | IDENTIFIER |
| --- | --- | --- |
| Antibodies |  |  |
| IKKα/β | Abcam | ab32041 |
| p-IKKα/β | Abcam | ab194528 |
| p65 | Proteintech | 10745-1-AP |
| p-p65 | CST | #3033S |
| mTOR | CST | 2972s |
| p-mTOR | CST | 5536s |
| NALP1 | Thermo Fisher Scientific | PA5-17275 |
| NLRC4 | Abcam | ab201792 |
| AIM2 | Abcam | ab180665 |
| GSDMD | Abcam | Ab219800 |
| IRF7 | Abcam | ab109255 |
| p-IRF7 | CST | 24129s |
| PCNA | Proteintech | 10205-2-AP |
| JAK1 | Proteintech | 66466-1-Ig |
| p-JAK1 | CST | #66245 |
| JAK2 | Abcam | ab108596 |
| p-JAK2 | Abcam | ab32101 |
| STAT1 | CST | #14994 |
| p-STAT1 | Abcam | ab109461 |
| IFNGR1 | Proteintech | 10808-1-AP |
| IFNGR2 | Proteintech | 10266-1-AP |
| GSK3β | Proteintech | 22104-1-AP |
| p-GSK3β (Ser9) | Proteintech | 67558-1-Ig |
| p-GSK3β (Tyr216) | Abcam | ab68476 |
| DRP1 | Abcam | ab156951 |
| OPA1 | Proteintech | 27733-1-AP |
| MFN1 | Proteintech | 13798-1-AP |
| MFN2 | Proteintech | 12186-1-Ap |
| TLR4 | Proteintech | 66350-1-AP |
| MyD88 | Proteintech | 23230-1-AP |
| TRAF6 | Abcam | ab94720 |
| TRIF | Abcam | ab180689 |
| TRAF3 | Abcam | ab239357 |
| SOCS1 | Proteintech | 25852-1-AP |
| ERK1/2 | CST | #4695 |
| p-ERK1/2 | CST | #4370 |
| HSF1 | Proteintech | 67189-1-Ig |
| p-HSF1 (Ser326) | Abcam | ab76076 |
| STAT6 | Abcam | ab32520 |
| p-STAT6 | Abcam | ab235591 |
| IL-1β | Santa Cruz | Sc-7884 |
| NLRP3 | Abcam | ab214185 |
| ASC | Santa Cruz | SC-514414 |
| β-Actin | Proteintech | 66009-1-Ig |
| ASC for immunofluorescence | Santa Cruz | SC-365611 |
| Caspase-1 for immunofluorescence | Proteintech | 22915-1-AP |
| NLRP3 for immunofluorescence | Abcam | Ab214185 |
| Anti-DNA for immunofluorescence | Sigma | CBL186 |
| HSP60 for immunofluorescence | Proteintech | 66041-1-Ig |
| IFNGR1 for immunofluorescence  IFNGR2 for immunofluorescence | Abcam  Santa cruz | ab95673  sc-12752 |
| Rab5 for immunofluorescence | CST | 3547 |
| Rab7 for immunofluorescence | CST | 9367s |
| Rab11 for immunofluorescence | CST | 5589s |
| Giantin for immunofluorescence | Abcam | ab37266 |
| HSF1 for CHIP | Abcam | ab52757 |
| Bacterial and Virus Strains |  |  |
| Bovine Pasteurellamultocida serotype A strain CQ2 (PmCQ2) | Provided by Yuanyi Peng (Southwest University) | N/A |
| Chemicals, Peptides, and Recombinant Proteins |  |  |
| Melatonin | Sigma | M5250 |
| Lipopolysaccharide (LPS) | Sigma | L2630 |
| Recombinant murine IFN-γ | PeproTech | 315-05 |
| Recombinant murine IL-4 | PeproTech | 214-14 |
| Recombinant murine M-CSF | PeproTech | 315-02 |
| Recomninant human IFN-γ | PeproTech | 300-02 |
| DAPI  Lipofectamine™ 3000  Opti MEM I | Beyotime  Invitrogen  Gbico | P0131  L3000008  31985070 |
| Lipofectamine™ 2000 | Invitrogen | 11668027 |
| Rotenone | Selleck | S2348 |
| DMM | Sigma | 136441 |
| Antimycin A | MCE | HY-105755 |
| Oligomycin | Selleck | S1478 |
| Rapamycin | Selleck | S1039 |
| PDTC | MCE | HY-18738 |
| Z-YVAD | Selleck | S8507 |
| C-A1 | Promega | C9451 |
| 2-NP | Absin | abs817422 |
| GLPG0634 | MCE | HY-18300 |
| CEP-33779 | MCE | HY-15343 |
| Fludarabine | MCE | HY-B0069 |
| MG132 | MCE | HY-13259 |
| CHX | Selleck | S7418 |
| CPZ | Selleck | S5749 |
| AMR | ApexBio | B1884 |
| NY | MCE | HY-17409 |
| SB216763 | MCE | HY-12012 |
| DMAT | MCE | HY-15535 |
| Compound C | MCE | HY-13418A |
| Poloxin | MCE | HY-12134 |
| SP600125 | MCE | HY-12041 |
| H-89 | MCE | HY-15979A |
| Luzindole | MCE | HY-101254 |
| 4-P-PDOT | MCE | HY-100609 |
| Prazosin | Selleck | S5780 |
| KN-93 | MCE | HY-15465 |
| SR1001 | MCE | HY-13421 |
| Nicotinamide | Sigma | N0636 |
| PMA (TPA) | Sigma | P1585 |
| SCH772984 | ApexBio | A3809 |
| Critical Commercial Assays |  |  |
| Murine IL-1β ELISA for cell culture supernatant | CUSABIO  Proteintech | CSB-E08054m  KE10003 |
| Murine IL-1β ELISA for serum and lung | Invitrogen | BMS6002TEN |
| Murine TNF-α ELISA for cell culture supernatant | Proteintech | KE10002 |
| Murine TNF-α ELISA for serum and lung | Invitrogen | BMS607-3TEN |
| Murine IL-10 ELISA for cell culture supernatant | Proteintech | KE10008 |
| Human IL-1β ELISA for cell culture supernatant | Proteintech | KE00021 |
| Mito-Tracker Red CMXRos | Beyotime | C1049B |
| Complex Ⅰ assay kit | Nanjing Jiancheng | A089-1-1 |
| Complex Ⅱ assay kit | Nanjing Jiancheng | A089-2-1 |
| Complex Ⅲ assay kit | Nanjing Jiancheng | A089-3-1 |
| Complex Ⅳ assay kit | Nanjing Jiancheng | A089-4-1 |
| Complex Ⅴ assay kit | Nanjing Jiancheng | A089-5-1 |
| CHIP kit | Abcam | ab500 |
| JC-1 kit | Beyotime | C2003S |
| ATP assay kit | Beyotime | S0026B |
| MitoSox | Invitrogen | M36008 |
| Fluo-4 AM | Beyotime | S1060 |
| Seahorse Extracellular Flux Analyzer XFe 24 | Agilent | www.agilent.com |
| Deposited Data |  |  |
| RNA-Seq | This Paper | PRJNA734047 |
| Experimental Models: Cell Lines |  |  |
| ANA-1 | Provided by Yuexia Liao (Yangzhou University) | N/A |
| THP-1 | Provided by Jielin Duan (Tongji University) | N/A |
| Experimental Models: Organisms/Strains |  |  |
| ICR mice | In-house colony | N/A |
| Oligonucleotides |  |  |
| siRNA for murine IRF7 (CCCUCUGCUUUCUAGUGAUTT)  siRNA for murine IFNGR2 (GAGACAAAGTGTGACTTAA)  siRNA for murine MT1  (CCTTAACAACGGATGGAAT) | Genecreate  Ribobio  Ribobio | N/A  N/A  N/A |
| siRNA for murine MT2  (GCCATTATCCGTGACGGTT) | Ribobio | N/A |
| siRNA for murine Hsf1  (GGATGATCCCACCATCTCT) | Ribobio | N/A |
| siRNA for murine Elk1  (#1 CAGCCATCCTAACAGAGAA)  (#2 CAGCCTGAGGTGTCTGTAA)  (#3 GGTACTACTATGATAAGAA) | Ribobio | N/A |
| siRNA for murine E2f1  (#1 GGATCTGGAGACTGACCAT)  (#2 GCAGAAACGGCGCATCTAT)  (#3 GGGTGAGGGCATTAGAGAT) | Ribobio | N/A |
| iNOS FW (CAGCTGGGCTGTACAAACCTT) | Sangon Biotech | N/A |
| iNOS RV (CATTGGAAGTGAAGCGTTTCG) | Sangon Biotech | N/A |
| IL-1β FW (GCAACTGTTCCTGAACTCAACT) | Sangon Biotech | N/A |
| IL-1β RV (ATCTTTTGGGGTCCGTCAACT ) | Sangon Biotech | N/A |
| mtATP6 FW (CAGTCCCCTCCCTAGGACTT) | Sangon Biotech | N/A |
| mtATP6 RV (TCAGAGCATTGGCCATAGAA) | Sangon Biotech | N/A |
| Rpl13a FW (GGGCAGGTTCTGGTATTGGAT) | Sangon Biotech | N/A |
| Rpl13a RV (GGCTCGGAAATGGTAGGGG) | Sangon Biotech | N/A |
| IRF7 FW (AGAGCGAAGAGGCTGGAAGACC) | Sangon Biotech | N/A |
| IRF7 RV(AGCATTGCTGAGGCTCACTTCTTC) | Sangon Biotech | N/A |
| IFNGR1 FW (GGCTCTGGAGGCTGGAGGAAG) | Sangon Biotech | N/A |
| IFNGR1 RV (TGATAGGCGGTGAGGCTACAAGG) | Sangon Biotech | N/A |
| IFNGR2 FW (CCAGCAATGACCCAAGACCAGTG) | Sangon Biotech | N/A |
| IFNGR2 RV (CAGTTCGGCTCCAGCAACCTATG) | Sangon Biotech | N/A |
| Cebpd FW (TCCTGCCATGTACGACGACGAG) | Sangon Biotech | N/A |
| Cebpd RV (CGCTTTGTGGTTGCTGTTGAAGAG) | Sangon Biotech | N/A |
| Elk1 FW (ATCCCTGCTCCCCACACATACC) | Sangon Biotech | N/A |
| Elk1 RV (GCTGCCACTGGACGGAAACTG) | Sangon Biotech | N/A |
| Junb FW (TGGCAGCGGTGGAGGTACAG) | Sangon Biotech | N/A |
| Junb RV (ACGTGGTTCATCTTGTGCAGGTC) | Sangon Biotech | N/A |
| Mafg FW (GCGAGAGTTGAACCAGCACCTG) | Sangon Biotech | N/A |
| Mafg RV (TTCTCCAGCTCCTCCTTCTGTGTC) | Sangon Biotech | N/A |
| Stat5a FW (AGTCGGTGACGGAGGAGAAGTTC) | Sangon Biotech | N/A |
| Stat5a RV (CGGTGGCAGTAGCATTGTGGTC) | Sangon Biotech | N/A |
| Hsf1 FW (CAAGGAGGTGCTGCCCAAGTAC) | Sangon Biotech | N/A |
| Hsf1 RV (CAAGGATGCTGGAACTCGGTGTC) | Sangon Biotech | N/A |
| Pou3f1 FW (CGTGTTCTCGCAGACCACCATC) | Sangon Biotech | N/A |
| Pou3f1 RV (CCTCCAGCCACTTGTTGAGCAG) | Sangon Biotech | N/A |
| Gata2 FW (CATGAAGATGGAAGGCGGCAGTC) | Sangon Biotech | N/A |
| Gata2 RV (GCGGGCACATAGGAGGGATAGG) | Sangon Biotech | N/A |
| Usf2 FW (GCTTCAGACAGGCACACAGAGG) | Sangon Biotech | N/A |
| Usf2 RV (TCCCTCCTTCTCCGTTCCACTTC) | Sangon Biotech | N/A |
| Stat4 FW (TGGGAGCCTCTCAGTGGAGTTTAG) | Sangon Biotech | N/A |
| Stat4 RV (CCATGTGGCAGCCCTCGTTTC) | Sangon Biotech | N/A |
| E2f1 FW (AGGAGAGTGCAGACGGGATTAGC) | Sangon Biotech | N/A |
| E2f1 RV (GGGATGTGGAGGGAGGTGATGG) | Sangon Biotech | N/A |
| Idh1 FW (GGGCGTCAAGTGTGCTACCATC) | Sangon Biotech | N/A |
| Idh1 RV (CTGAAGACAGTGCCACCCAGAATG) | Sangon Biotech | N/A |
| Idh2 FW (TGTGCTGGTCTGCCCTGATGG) | Sangon Biotech | N/A |
| Idh2 RV (GCCCTTCTGGTGTTCTCGGTAATG) | Sangon Biotech | N/A |
| Idh3a FW (TGCTGGTGGTGTTCAGACAGTAAC) | Sangon Biotech | N/A |
| Idh3a RV (CCTCCTGGTCCTTGAATTGCTGTG) | Sangon Biotech | N/A |
| Idh3g FW (CCTCTATGCCAACGTCATCCACTG) | Sangon Biotech | N/A |
| Idh3g RV (GCTGCTGTACTCGCCTTCTGTG) | Sangon Biotech | N/A |
| Sdhd FW (AGCACATTCACCTGTCACCAAGC) | Sangon Biotech | N/A |
| Sdhd RV (CCACCACAGAGCAGGGATTCAAG) | Sangon Biotech | N/A |
| Got2 FW (CCAGCGGTGATGGTGATAAGGATG) | Sangon Biotech | N/A |
| Got2 RV (ACGATTGGCAGAGGCAGACATTG) | Sangon Biotech | N/A |
| Gls FW (GTCTGCCCTCCGAAGGTTTGC) | Sangon Biotech | N/A |
| Gls RV (CCTCTGCTGCTGCGACATGG) | Sangon Biotech | N/A |
| Gs FW (TCCACGAAACCTCCAACATCAACG) | Sangon Biotech | N/A |
| Gs RV (CGACGGTCTTCAAAGTAGCCCTTC) | Sangon Biotech | N/A |
| Glud FW (GCATCTTGGAGGCTGACTGTGAC) | Sangon Biotech | N/A |
| Glud RV (TGACTCTGGGTGCATTGGATTTGG) | Sangon Biotech | N/A |
| Fh1 FW (ACTCAGTGTGAAGCGATGACCATG) | Sangon Biotech | N/A |
| Fh1 RV (ATGTCCATTGCTGCCTCCAACG) | Sangon Biotech | N/A |
| Mdh1 FW (GCCATCGCAGACCACATCAGAG) | Sangon Biotech | N/A |
| Mdh1 RV (GGACACCATAGGAGTTGCCATCAG) | Sangon Biotech | N/A |
| Aco2 FW (AGGTGGGTGGTGATTGGAGATGAG) | Sangon Biotech | N/A |
| Aco2 RV (CCTGGCGAAGCTCTTGGTGATG) | Sangon Biotech | N/A |
| MT1 FW (ACCGCAACAAGAAGCTCAGGAAC) | Sangon Biotech | N/A |
| MT1 RV (GATGTCAGCACCAAGGGATAAGGG) | Sangon Biotech | N/A |
| MT2 FW (CCTCGTCTGGCTCCTCACTCTG) | Sangon Biotech | N/A |
| MT2 RV (GTGTGCTGGCTGTCTGGATGAAG) | Sangon Biotech | N/A |
| Ifngr2 (Chip) FW (AGCTACCGGACACTTCCCTATGTG) | Sangon Biotech | N/A |
| Ifngr2 (Chip) RV (GCCCTCTTAACTGCTGAGCGAATC) | Sangon Biotech | N/A |
| GAPDH FW (AGGTCGGTGTGAACGGATTTG) | Sangon Biotech | N/A |
| GAPDH RV (TGTAGACCATGTAGTTGAGGTCA) | Sangon Biotech | N/A |
| β-actin FW (AACAGTCCGCCTAGAAGCAC) | Sangon Biotech | N/A |
| β-actin RV (CGTTGACATCCGTAAAGACC) | Sangon Biotech | N/A |
| Recombinant DNA |  |  |
| Plasmid: pcDNA3.1(+)-Mam-Irf7 | Genecreate | N/A |
| Plasmid: pcDNA3.1(+)-Mam-Ifngr2 | Genecreate | N/A |
| Plasmid: pcDNA3.1(+)-Mam-Hsf1 | Genecreate | N/A |
| Software and Algorithms |  |  |
| Wave Software Version 2.3 | Agilent | www.agilent.com |
| FlowJo v10  ImageJ | FlowJo  NIH | www.flowjo.com  www.imagej.nih.gov |
| ZEN 2 | Zeiss | www.zeiss.com |
| Graphpad Prism 8 | GraphPad Software | www.graphpad.com |

**Supplementary figure legends**

**Fig. S1. Effects of melatonin on inflammatory responses of LPS/IFN-γ-stimulated or IL-4-stimulated macrophages. (A)** Proliferation of PEMs with the treatments as indicated by CCK-8 method [LPS (1 μg/mL) plus IFN-γ (20 ng/mL) with or without melatonin (1 mM) for 12 h, the same as below unless indicated] (n=6). Results represent three independent experiments. **(B and C)** Flow cytometry analysis of the total apoptosis of PEMs (n=4). **(D and E)** Go analysis **(D)** and KEGG analysis **(E)** of DEGs in macrophages (n=4). **(F)** The release of active caspase-1 p10 and mature IL-1β in culture supernatants and protein abundance of GSDMD in lysate of PEMs (n=3). **(G-I)** The secretion of IL-10 **(G)** and protein abundance of STAT6 and p-STAT6 **(H and I)** in PEMs with treatments as indicated [IL-4 (20 ng/mL) with or without melatonin (1 mM) for 12 h] (n=3-4). Data were analyzed with unpaired t-test (**A**, **C**, and **I**) or one-way ANOVA with Bonferroni correction (**G**) and represented as means ± SD. **P*< 0.05, ****P*< 0.001.

**Fig. S2. Effects of melatonin on mitochondrial functions and cellular metabolism of LPS/IFN-γ-stimulated macrophages. (A-D)** Mitochondrial membrane potential **(A)**, intracellular ATP level **(B)**, mtROS level **(C)**, and cytosolic Ca^2+^ level **(D)** of PEMs with treatments as indicated (n=3-4). Results represent two independent experiments. **(E)** The secretion of IL-1β from PEMs with treatments as indicated [in some group, NAC (5 mM) was pre-treated for 2 h] (n=3). **(F and G)** Protein abundance of DRP1, OPA1, MFN1, and MFN2 in macrophages (n=3). Results represent two independent experiments. **(H)** Heatmap analysis of DEGs enriched in TCA cycle in PEMs with treatments as indicated (n=4). **(I)** Relative mRNA expression of Idh1, Idh2, Idh3a, and Idh3g in macrophages (n=3). Data shown as means ± SEM. **(J and K)** Principal Component Analysis (PCA) of PEMs with treatments as indicated by 200MRM method **(J)** and GC-TOF-MS method **(K)**(n=3). **(L)** Heatmap analysis of different metabolites in macrophages by GC-TOF-MS method (n=6). **(M)** Fold change of Got2, GLS, GS, Glud, Fh1, Mdh1, Cs, and Aco2 in macrophages (n=4). Data were analyzed with unpaired t-test (**M**) or one-way ANOVA with Bonferroni correction (**A-E**, **G,** and **I**) and represented as means ± SD unless indicated. **P*< 0.05, ***P*< 0.01, ****P*< 0.001, *****P*< 0.0001.

**Fig. S3. IRF7 is the hub gene but is not associated with the expression of metabolic enzymes in LPS/IFN-γ-stimulated macrophages. (A)** Venn diagram of DEGs enriched in cellular pathways and Top 30 DEGs with high kWithin value by Connectivity analysis. **(B)** Heatmap analysis of the genes selected based on **A**. **(C)** Relative mRNA expression of IRF7 in PEMs with treatments as indicated (si-IRF7: IRF7 silencing; IRF7 OE: IRF7 overexpression, the same as below unless indicated) (n=3). Results represent two independent experiments. **(D-G)** Protein abundance of IRF7 and p-IRF7 in macrophages with treatments as indicated (n=4). Data shown as means ± SD. **(H)** The secretion of IL-1β from PEMs with treatments as indicated (n=3). Data shown as means ± SD. **(I)** Relative mRNA expression of Idh1, Idh2, Idh3a, Idh3g, Sdhd, Got2, Gls, Gs, Glud, Fh1, Mdh1, and Aco2 in macrophages with treatments as indicated (n=3). Data were analyzed with unpaired t-test (**E** and **G**) or one-way ANOVA with Bonferroni correction (**H** and **I**) and represented as means ± SEM unless indicated. **P*< 0.05, ***P*< 0.01, ****P*< 0.001, *****P*< 0.0001.

**Fig. S4. TLR4-MyD88-TRAF6 and TLR4-TRIF-TRAF3 pathways do not contribute to the reduced IL-1β production in macrophages upon melatonergic activation. (A and B)** Protein abundance of TLR4, MyD88, TRAF6, TRIF, TRAF3, IRF7, and p-IRF7 in LPS/IFN-γ-stimulated PEMs with or without melatonin treatment at indicated time points (n=3). **(C and D)** Protein abundance of TLR4, MyD88, TRAF6, TRIF, and TRAF3 in LPS/IFN-γ-stimulated PEMs with or without melatonin treatment at indicated time point (n=3). **(E)** Protein abundance of SOCS1 in PEMs with treatments as indicated (n=3). **(F)** The secretion of IL-1β from macrophages with treatments as indicated (in some groups, pre-treated with GLPG0634: 10 μM, CEP-33779: 1 μM, Fludarabine: 10 μM, for 1 h) (n=3). Results represent three independent experiments. **(G)** Protein abundance of IFNGR2 in macrophages with treatments as indicated (si-IFNGR2: IFNGR2 silencing; IFNGR2 OE: IFNGR2 overexpression) (n=3). Data were analyzed with one-way ANOVA with Bonferroni correction (**B**, **D**, **E**, right panel, **F**, and **H**) and represented as means ± SD. **P*< 0.05, ***P*< 0.01, ****P*< 0.001, *****P*< 0.0001.

**Fig. S5. Effects of melatonin on IFNGR2 expression in LPS/IFN-γ-stimulated macrophages. (A)** Heatmap analysis of initiation factors, elongation factors, termination factors, and translational repressor in macrophages (n=4), up-regulated (red) and down-regulated (blue). **(B)** Relative mRNA expression of Elk1 and E2f1 in macrophages with treatments as indicated (si-Elk1: Elk1 silencing; si-E2f1: E2f1 silencing) (n=3). Results represent two independent experiments. **(C)** The secretion of IL-1β from PEMs with treatments as indicated in **(B)** (n=3). Results represent two independent experiments. Data shown as means ± SD. **(D)** Relative mRNA expression of Hsf1 in macrophages with treatments as indicated (si-HSF1: Hsf1 silencing; HSF1 OE: Hsf1 overexpression) (n=3). **(E)** Promoter sequence of *Ifngr2* by UCSC program. Data were analyzed with unpaired t-test (**D**) or one-way ANOVA with Bonferroni correction (**B** and **C**) and represented as means ± SEM unless indicated. **P*< 0.05, ***P*< 0.01, ****P*< 0.001.

**Fig. S6. p-ERK1/2 and p-HSF1 (Ser326) are not involved in melatonin-mediated suppressive effect on LPS/IFN-γ-stimulated macrophage inflammation. (A)** The secretion of IL-1β from PEMs with treatments as indicated [in some groups, Luzindole (10 μM), 4-P-PDOT (50 μM), Prazosin (1.5 μM), KN-93 (1 μM), SR1001 (10 μM), was respectively added with melatonin] (n=3). **(B)** The secretion of IL-1β from PEMs with treatments as indicated [in some group, Nicotinamide (5 mM) was added with melatonin] (n=3). **(C and D)** Relative mRNA expression of MT1 **(C)** and MT2 **(D)** in macrophages with treatments as indicated (si-MT1: MT1 silencing; si-MT2: MT2 silencing, the same as below unless indicated) (n=3) Results represent three independent experiments. Data shown as means ± SEM. **(E)** Protein abundance of ERK1/2 and p-ERK1/2 in macrophages with treatments as indicated (n=3). **(F)** The secretion of IL-1β from macrophages with treatments as indicated [in some groups, TPA (2 μM) or SCH772984 (2 μM) was added without or with melatonin, respectively] (n=3). **(G and H)** Protein abundance of HSF1 and p-HSF1 (Ser326) **(G)** and GSK3β and p-GSK3β (Tyr216) **(H)** in macrophages with treatments as indicated (n=3). Data were analyzed with unpaired t-test (**C**, and **D**) or one-way ANOVA with Bonferroni correction (**A**, **B**, **E**, right panel, **F**, **G**, right panel, and **H**) and represented as means ± SD unless indicated. **P*< 0.05, ***P*< 0.01, ****P*< 0.001.

**Fig. S7. Melatonin reduces levels of *Pasteurella multocida* infection-induced proinflammatory cytokines in mice. (A and B)** Levels of IL-1β **(A)** and TNF-α **(B)** in serum of PmCQ2-infected mice pre-treated with or without melatonin (30, 60, or 120 mg/kg BW) at 16 h, 24 h, and 32 h post infection (n=6-8), the same as below unless indicated. **(C and D)** Relative mRNA expression of IL-1β **(C)** and TNF-α **(D)** in lungs of PmCQ2-infected mice (n=6-8). Data shown as means ± SEM. **(E and F)** Levels of IL-1β **(E)** and TNF-α **(F)** in lungs of PmCQ2-infected mice (n=6-8). Data were analyzed with one-way ANOVA with Bonferroni correction (**A-F**) and represented as means ± SD unless indicated. **P*< 0.05, ***P*< 0.01, ****P*< 0.001, *****P*< 0.001.
