# Supplementary figures and images for: Melatonergic signalling instructs transcriptional inhibition of IFNGR2 to lessen interleukin‐1β‐dependent inflammation
Source: Clin Transl Med. 2022 Feb 20;12(2):e716. doi: 10.1002/ctm2.716 (PMC8858632; doi:10.1002/ctm2.716)

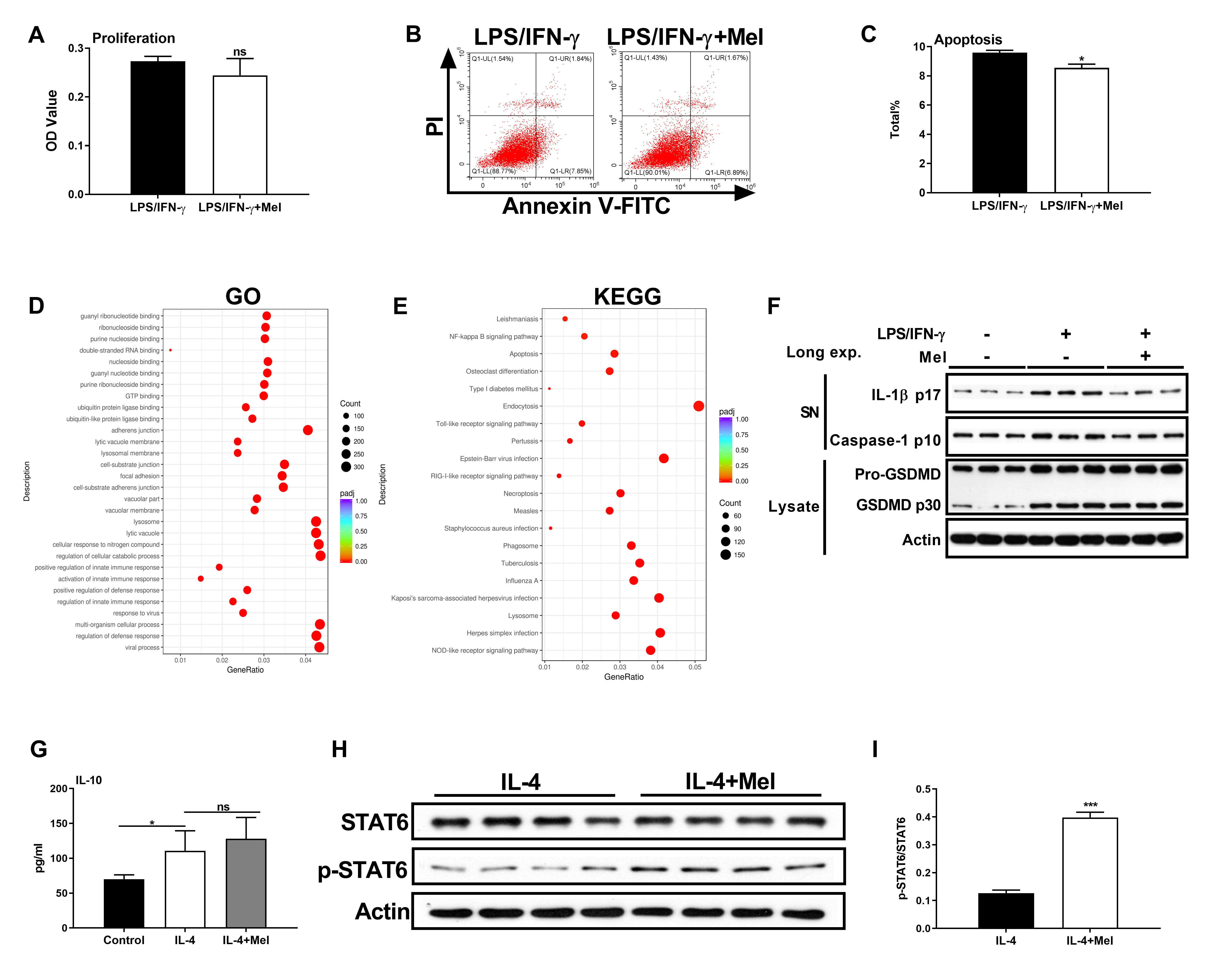

Supplement: Supplementary file 2 — Supporting information [file CTM2-12-e716-s005.tif]

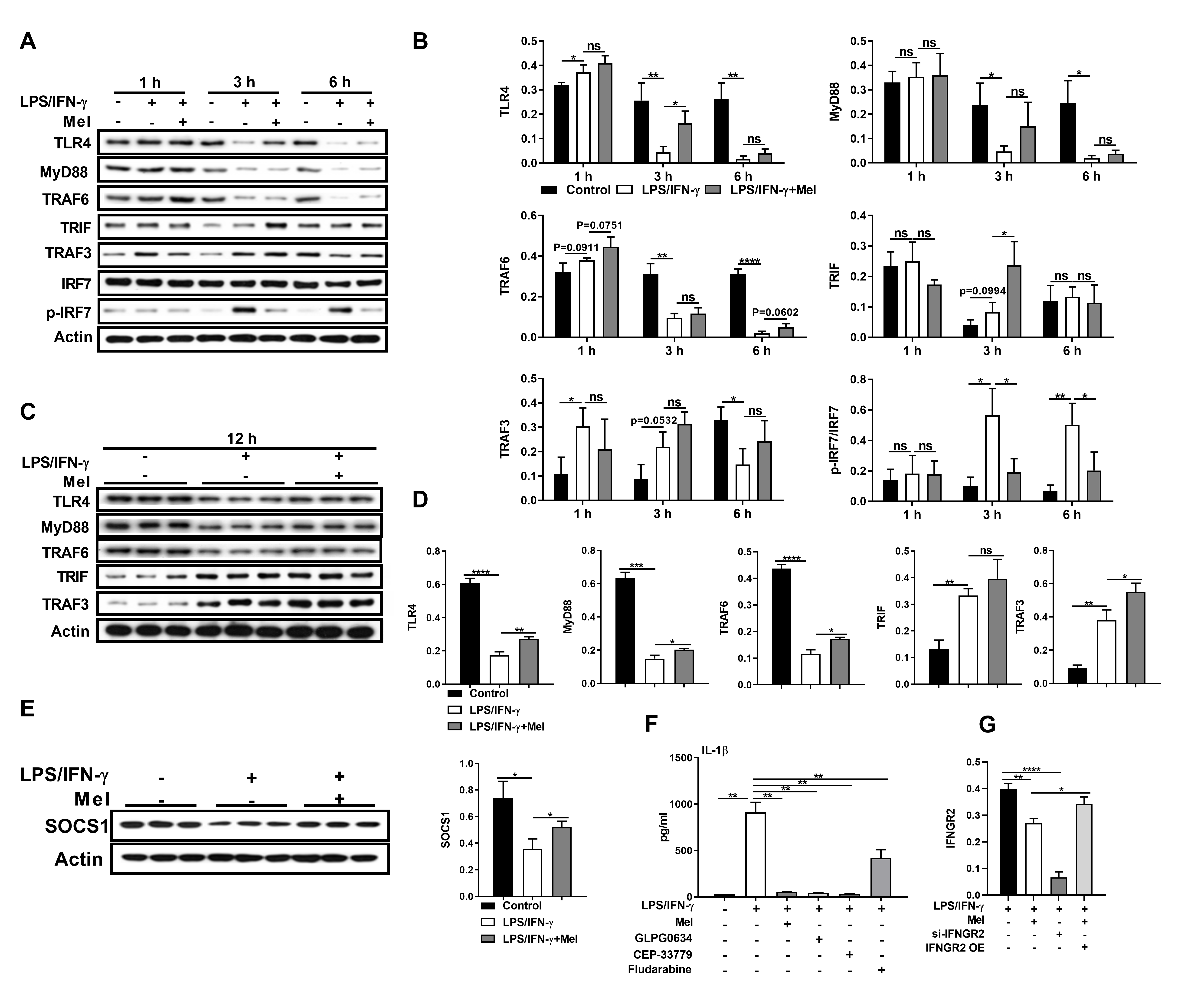

Supplement: Supplementary file 5 — Supporting information [file CTM2-12-e716-s004.tif]

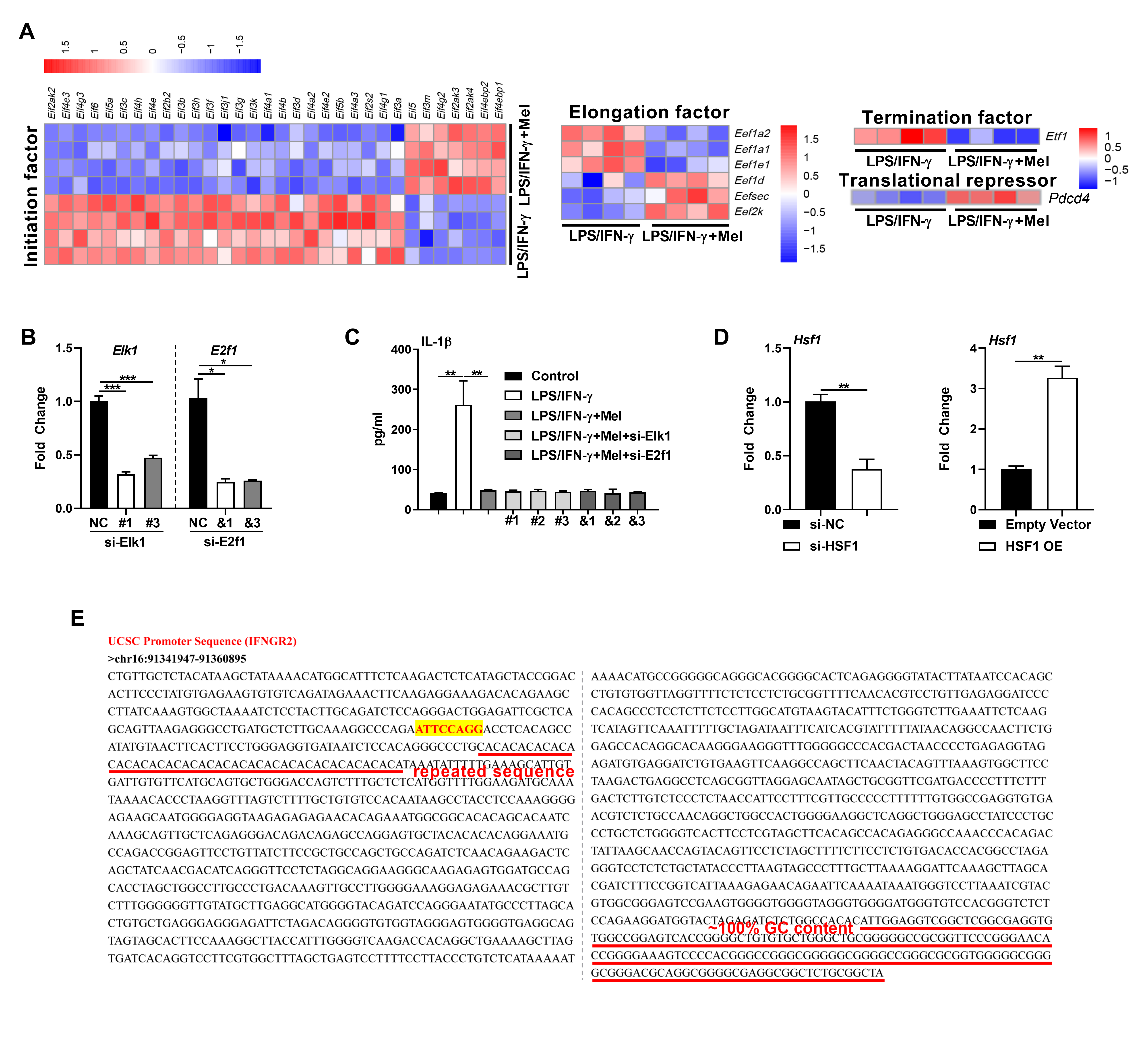

Supplement: Supplementary file 6 — Supporting information [file CTM2-12-e716-s002.tif]

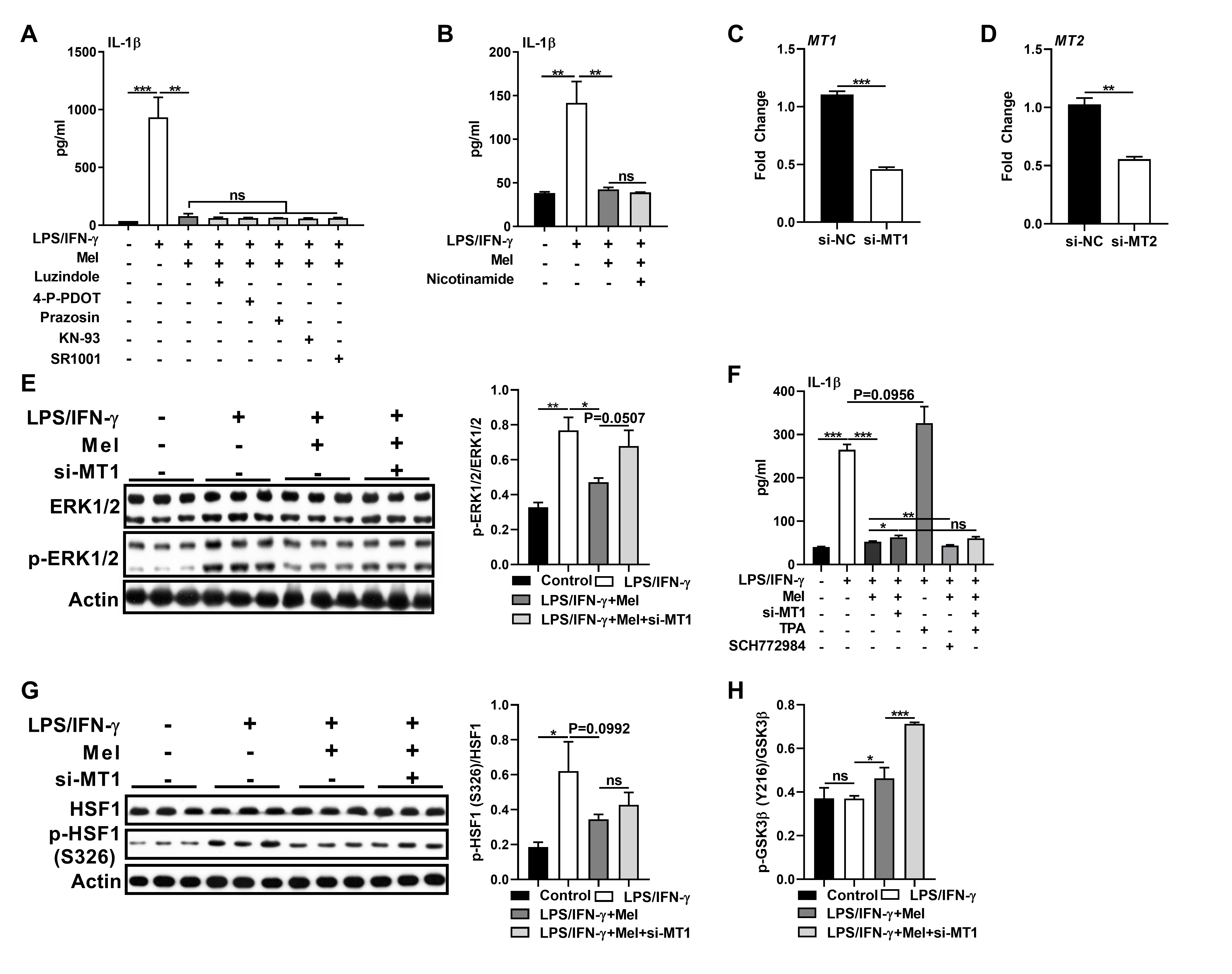

Supplement: Supplementary file 7 — Supporting information [file CTM2-12-e716-s007.tif]

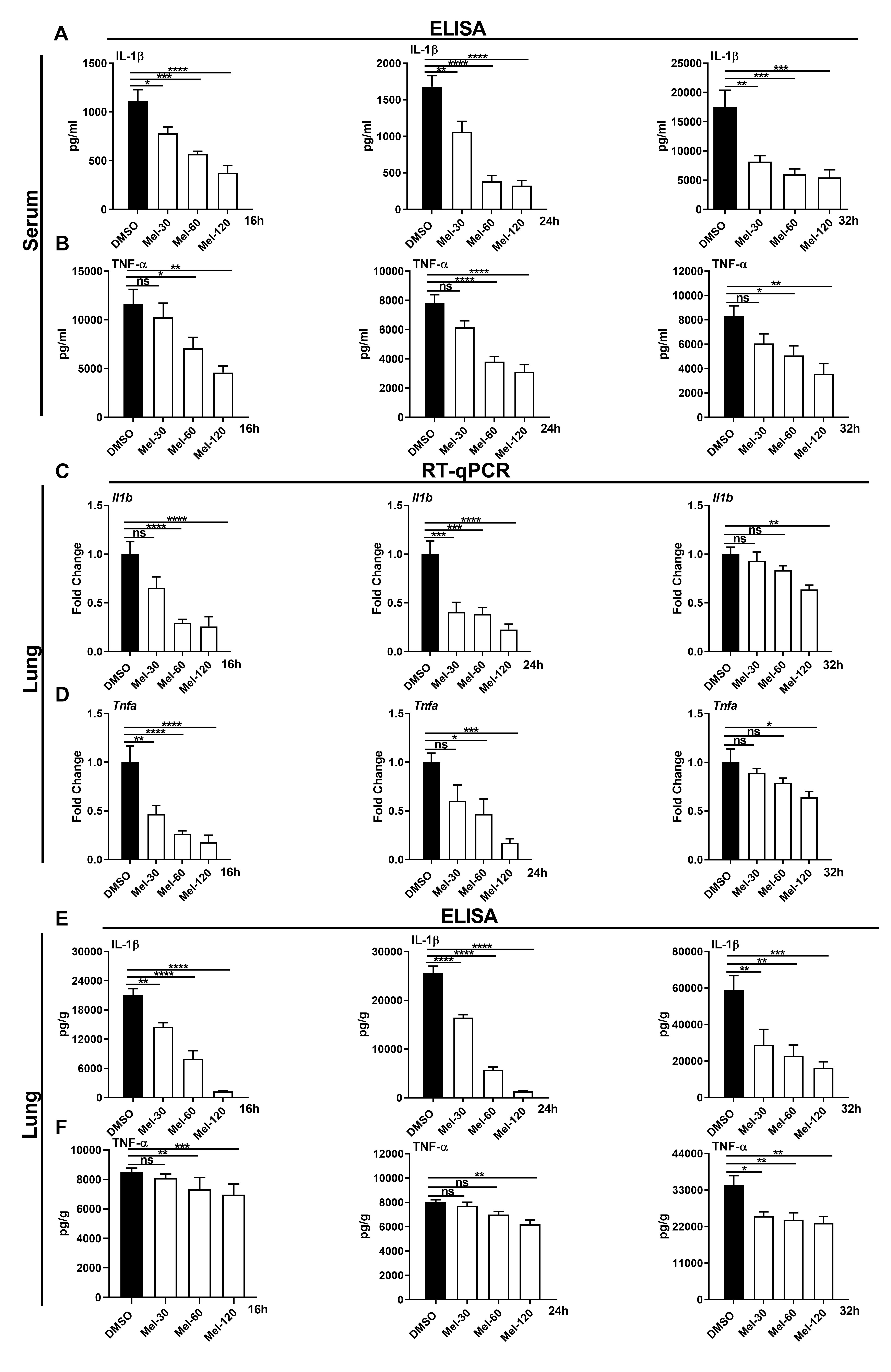

Supplement: Supplementary file 8 — Supporting information [file CTM2-12-e716-s008.tif]
